# Spatiotemporal Raman probing of molecular transport in sub–2-nm plasmonic quasi-2D nanochannels

**DOI:** 10.1126/sciadv.aec3641

**Published:** 2026-02-25

**Authors:** Haoran Liu, Zihe Jiang, Zhiwei Hu, Banghuan Zhang, Tao He, Xiaohui Dong, Chaowei Sun, Jun Tian, Wei Jiang, Ferruccio Pisanello, Huatian Hu, Wen Chen, Hongxing Xu

**Affiliations:** ^1^ State Key Laboratory of Precision Spectroscopy, East China Normal University, Shanghai 200241, China.; ^2^ Institute of Laser Manufacturing, Henan Academy of Sciences, Zhengzhou 450046, China.; ^3^ Department of Chemistry, State Key Laboratory of Porous Materials for Separation, and Conversion, Fudan University, Shanghai 200438, China.; ^4^ Istituto Italiano di Tecnologia, Center for Biomolecular Nanotechnologies, Via Barsanti 14, 73010 Arnesano, Italy.

## Abstract

Capturing molecular dynamics in nanoconfined channels with high spatiotemporal resolution is a key challenge in nanoscience, crucial for advancing catalysis, energy conversion, and molecular sensing. Bottom-up ultrathin plasmonic nanogaps, such as nanoparticle-on-mirror (NPoM) structures, are ideal for ultrasensitive probing due to their extreme light confinement, but their perceived sealed geometry has cast doubt on the existence of accessible transport pathways. Here, counterintuitively, we demonstrate that ubiquitous ligand-capped NPoM-type nanogaps can form a natural quasi–two-dimensional nanochannel, supporting molecular exchange and infiltration over unprecedented length scales (≳5 micrometers) with an extreme aspect ratio (>10^3^). Using wavelength-multiplexed Raman spectroscopy, we resolve the underlying centripetal infiltration pathway with a spatial resolving power of ~20 nanometers. This redefines the NPoM architecture as a sensitive and hotspot-accessible platform, enabling in situ, real-time, reusable monitoring of analyte with ~10^−11^ molar. This work establishes a versatile platform for advancing super-resolved in situ molecular sensing, nanoscale physicochemical studies, and on-chip nanophotofluidics.

## Teaser

Seemingly sealed plasmonic nanogaps act as accessible channels for real-time, ultrasensitive molecular sensing.

## INTRODUCTION

Probing and understanding molecular dynamics within nanoconfined environments are fundamental to surface physical chemistry and nanoscience (*1*–*4*). Confinement within low-dimensional geometries—such as zero-dimensional (0D) nanopores (*3*–*5*), 1D nanotubes (*6*), and 2D nanochannels (*7*, *8*)—imposes physical constraints that radically alter molecular transport, reactivity, and selectivity compared to bulk systems, giving rise to important applications ranging from molecular sieving to ultrafast and dynamically tunable transport (*5*–*10*). While these phenomena can be characterized by various means including electrical readouts (*6*, *9*, *10*) and structural imaging (*7*), optical methods offer particularly powerful, noninvasive routes for tracking molecular behavior in real time (*3*, *4*). However, conventional optics are restricted by the diffraction limit and intrinsically weak light-matter interactions, creating a critical need for effective approaches that provide label-free, single-molecule sensitivity with high spatiotemporal resolution.

Plasmonic nanostructures can overcome this challenge by concentrating light into deep subwavelength volumes, thereby greatly enhancing light-matter interactions. Recent advances in plasmonics have ushered in an era of extreme nanophotonics (*11*–*14*), where metallic nanogaps can trap light into a 1-nm^3^ volume, achieving atomic-scale sensitivity (*15*, *16*), opening doors for vast key applications such as single-molecular science (*17*–*21*) and surface chemistry (*19*, *22*–*25*). The integration of plasmonics with nanofluidics has yielded powerful analytical platforms (*3*, *4*). A prominent example is the plasmonic nanopore, typically fabricated via top-down methods, which funnels analytes through electrophoresis (*26*) or optical trapping (*27*) for applications such as DNA and protein analysis (*26*–*31*). These systems enable real-time characterization through signals such as fluorescence enhancement (*28*), resonance shift (*29*), or surface-enhanced Raman spectroscopy (SERS) (*26*, *31*). However, the 0D nature of nanopores inherently limits analyte dwell time, which hinders the detection of weak or specific signals and the observation of subtle or slow dynamic processes (*3*, *4*). Furthermore, the top-down fabrication of reliable, sub–5-nm gaps remains a considerable and costly challenge—a limitation shared by other top-down nanogap configurations that often suffer from poor geometric control (*32*). In contrast, 2D nanochannels offer an extended spatial dimension, providing a much longer observation window ideal for studying molecular infiltration and interaction dynamics. However, achieving high-spatiotemporal-resolution probing of molecular dynamics in these 2D plasmonic nanochannels remains challenging.

Among plasmonic architectures, the nanoparticle-on-mirror (NPoM) geometry stands out as a highly tunable, high-quality-factor, and reproducible nanoplatform (*11*, *12*, *33*). Formed through simple, high-yield bottom-up assembly, it offers well-defined and consistent gap dimensions toward a subnanometer scale that supports huge field enhancement. Intuitively, the insulating layer that mechanically supports the nanoparticle in this vertically assembled metal-insulator-metal nanogap (*12*) would appear to block and seal the 2D nanochannel formed between the two metallic surfaces in the gap. Consequently, their application has been predominantly focused on probing pre-embedded analytes—such as biomolecules, self-assembled monolayers (SAMs), or 2D materials (*19*, *20*, *33*–*35*)—or has been limited to leveraging only the edge regions of the nanogap for postsensing (*36*, *37*), rather than exploiting the central hotspot as an open platform for dynamic sensing (*38*). Parallel efforts to engineer open bottom-up nanogaps for molecular access and transport have been pursued (*20*, *39*, *40*), for example, through host-guest chemistry (*20*) and metal-organic frameworks (MOFs) (*40*). Additional postfabrication methods such as surface cleaning (*41*, *42*), ligand exchange (*23*, *43*–*48*), and electrochemical cycling (*49*–*51*) have been used to reuse the plasmonic surfaces or gaps. However, these studies have largely focused on the ensemble-averaged effects of static adsorption or ex situ detection. The real-time, spatially resolved observation of molecular infiltration dynamics within an individual gap has remained unaddressed. This creates a clear division: Top-down structures with open channels allow postfabrication sensing but suffer from limited performance, while bottom-up systems such as NPoM offer superior optical properties but are often treated as closed, or their internal analyte dynamics remain a black box.

**Fig. 1.**
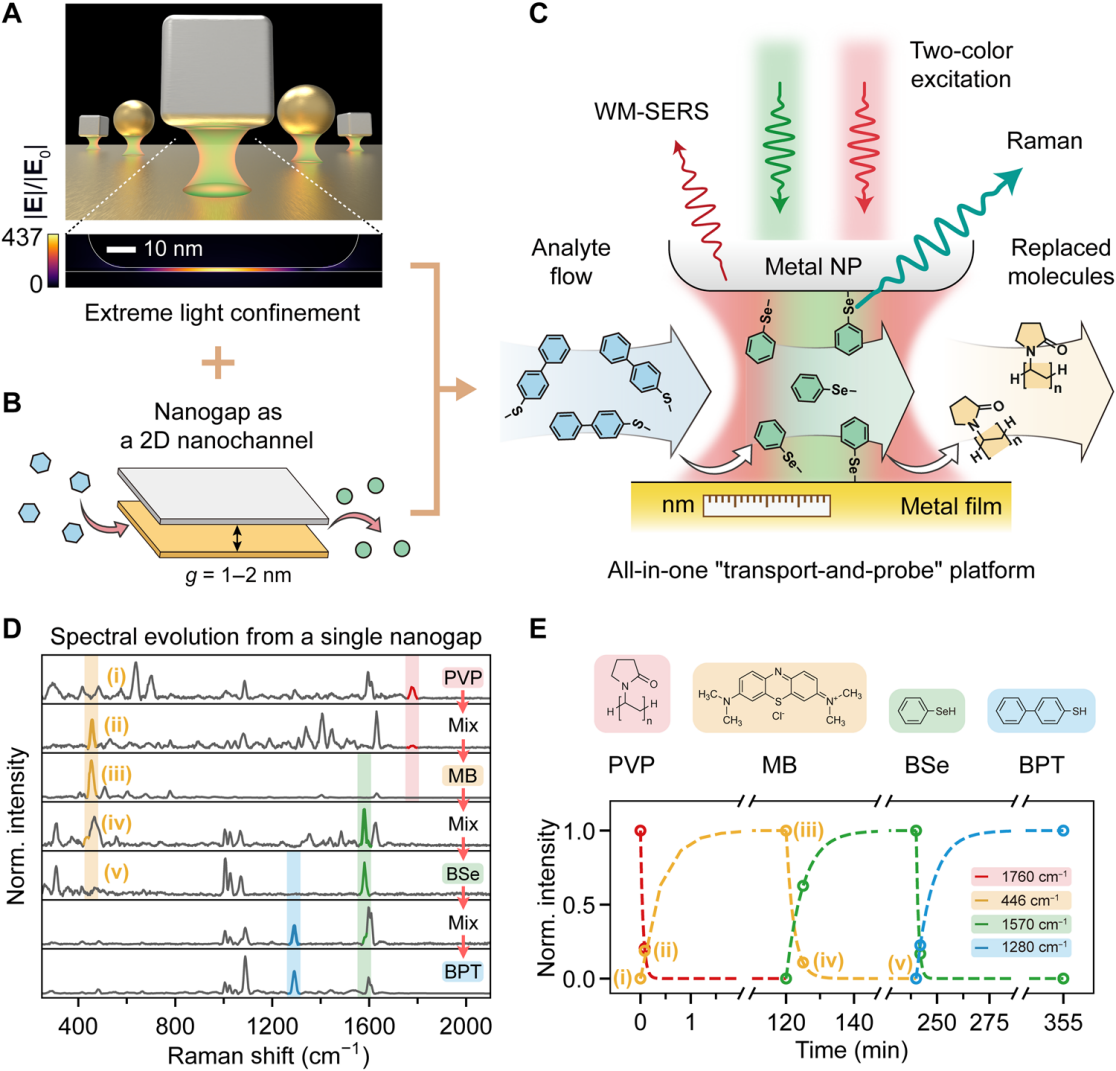
Sequential molecule exchange SERS sensing in NPoM plasmonic nanochannels. (**A**) Top: 3D schematics of metal nanoparticles (nanosphere, nanocube, etc.) placed over a metal mirror to form well-defined plasmonic nanogaps. Bottom: Simulated field distribution of a nanocube on a mirror (NCoM) with 1-nm-thick gap, exhibiting pronounced localized field enhancement ($|E|/|E_{0}|=437$). (**B**) Schematics of a molecule exchanges in a 2D molecular nanochannel with gap thickness *g* = 1 to 2 nm. (**C**) Schematic illustration of an all-in-one transport-and-probe platform using super-resolved WM-SERS spectroscopy for sensing of sequential molecular exchange dynamics in 1- to 2-nm-thick quasi-2D plasmonic nanochannels. NP, nanoparticle. (**D**) Normalized SERS spectra (excitation, 785 nm; integration time, 10 s) of a single PVP-capped NCoM before and after the sequential immersion in the solution of MB → BSe → BPT (from top to bottom). The characteristic peaks of each molecule are highlighted in red, yellow, green, and blue areas, respectively. (**E**) Molecular structures of PVP, MB, BSe, and BPT (top) and their corresponding characteristic SERS peak intensities [colored open circles, extracted from (D)] as a function of immersion duration, where the dashed lines are the fitting results by Langmuir kinetic equation (see details in the Supplementary Materials).

In this work, counterintuitively, we demonstrate that the simplest and ubiquitous molecular-spaced plasmonic nanogaps naturally host quasi-2D nanochannels, which can support molecular interactions and transports, spatiotemporally resolved using wavelength-multiplexed SERS (WM-SERS) (*52*, *53*). By harnessing the spatially complementary near-fields of distinct plasmonic modes, our WM-SERS technique achieves a spatial resolving power of ~20 nm. In the end, we show how the NPoM-type configuration can be redefined as an all-in-one nanoplatform (Fig. 1, A to C), where nanoconfined optical field overlaps with a natural functional nanochannel in the nanogap to “transport-and-probe” in situ molecular science. This natural quasi-2D nanochannel enables nanoconfined analyte infiltration via molecular exchange dynamics, for which we establish a mechanical model revealed by WM-SERS: Target molecules progressively diffuse from the nanogaps’ edge to central regions, achieving maximal SERS via a ligand-exchange mechanism, whose efficiency is dependent on molecular binding affinities. Furthermore, this molecular infiltration and exchange process can occur over unprecedented length scales (≳5 μm) within tightly confined (~2 nm) nanochannels, with an extreme aspect ratio of >10^3^, whose trajectory can be traced by SERS mapping on microplate-on-foil (MPoF) nanogaps. In addition, we further demonstrate that this platform enables real-time monitoring of nanoconfined molecular transport with microfluidic on-chip integration, where molecular exchange, etching, and transport occur on a timescale of seconds. Individual NPoM channels can achieve high sensitivity (10^−11^ M) for nonresonant small analytes toward single-molecule level using state-of-the-art digital SERS techniques. Our work introduces a versatile platform that merges extreme optical confinement with active mass transport, facilitating applications for super-resolved molecular sieving and sensing, nanoreactors, confined-phase chemistry, and nanophotofluidics.

## RESULTS

### Sequential molecule exchange in a single plasmonic nanogap

We begin by monitoring molecular exchange dynamics inside the well-defined 1- to 2-nm NPoM-type nanogap through SERS measurements to validate its role as a quasi-2D nanochannel. Fabrication of the plasmonic nanogaps was achieved via direct deposition of chemically synthesized colloidal nanoparticles onto a gold film (see details in Materials and Methods). The nanogap acts as an optical nanocavity providing extreme light confinement (enhancement compared with free-space incidence |**E**|/|**E**_0_| ≃ 437; Fig. 1A), while simultaneously serving as a quasi-2D molecular nanochannel that enables molecular transports and interactions inside (Fig. 1B). The surfactant ligand layers inherent to these nanoparticles—e.g., citrate, polyvinylpyrrolidone (PVP), or cetyltrimethylammonium chloride (CTAC)—function as 1- to 2-nm-thick molecular spacers, creating well-defined nanochannels conducive to controlled analyte infiltration and exchange in subsequent sensing applications. To verify this infiltration and exchange mechanism in the nanochannel, we use single-color SERS to probe the molecular components and two-color excitation WM-SERS to spatiotemporally resolve their exchange dynamics within a single NPoM.

We first used a single 70-nm PVP-capped silver nanocube on a mirror (NCoM). This single NCoM underwent sequential incubation cycles in solutions of methylene blue (MB), benzeneselenol (BSe), and biphenyl-4-thiol (BPT), using the PVP layer as the initial molecular spacer. Before and after each incubation step, single-nanoparticle SERS was performed using 785-nm laser excitation to monitor the molecular exchange dynamics in time within the same single nanogap (nanochannel). The resultant spectral evolution of the complete replacement sequence (PVP → MB → BSe → BPT) is shown in Fig. 1D. Upon immersion in MB for merely 5 s, characteristic PVP Raman peaks (red shade in Fig. 1D) were partially replaced by mixed spectral signatures of both PVP and MB. Extended incubation (2 hours) led to near-complete molecular exchange, with SERS spectra dominated exclusively by MB vibrational modes (yellow shade in Fig. 1D). Subsequent incubations in BSe and BPT solutions exhibited similar spectral changes, confirming efficient replacement of the preadsorbed molecular layers inside and outside the nanogaps. Figure 1E quantifies this multistep replacement dynamics, showing the normalized intensities (see the “Raman peak intensity extraction” section in Materials and Methods) of representative vibrational bands (demonstrated by colored regions in Fig. 1D) against incubation time. Taking the lifecycle of the MB molecule as an example, the curve traces the complete five-stage process (the five yellow open circles from left to right in Fig. 1E): (i) its initial absence, (ii) the partial and then (iii) saturated replacement of the preceding PVP layer, (iv) its subsequent partial replacement by the incoming BSe molecules, and, lastly, (v) its complete replacement from the nanochannel. This analysis, performed on the same single nanogap, provides a qualitative insight into the continuous molecular exchange occurring within the nanochannel.

To describe the temporal evolution of the molecular exchange, we applied a simplified Langmuir kinetic model to our experimental data (see details in the Supplementary Materials) (*54*). The fits, shown as dashed lines in Fig. 1E, effectively model the observed saturation dynamics. The extracted phenomenological rate parameters reveal a clear trend: The replacement of the weakly bound PVP spacer is exceptionally rapid, whereas the final exchange between BSe and BPT, two molecules with comparable binding affinities to gold, is the slowest step. This kinetic analysis provides a qualitative insight into the exchange process, highlighting that the platform can distinguish between analytes based on their apparent dynamic binding properties at the nanoscale. This analysis validates the proposed mechanism and highlights the platform’s capability to differentiate between analytes based on their dynamic binding properties at the nanoscale. Notably, this molecular infiltration is not strictly unidirectional or dictated solely by binding affinity. We found that even molecules with weaker binding energies, such as MB, can progressively replace the strongly bound BPT in the nanogap, albeit at a slower rate (see fig. S18). This demonstrates the bidirectional molecular infiltration capability of the nanochannel, underscoring its nature as a truly open and versatile platform.

### Spatiotemporally resolved molecular infiltration and exchange dynamics in individual nanogaps

**Fig. 2.**
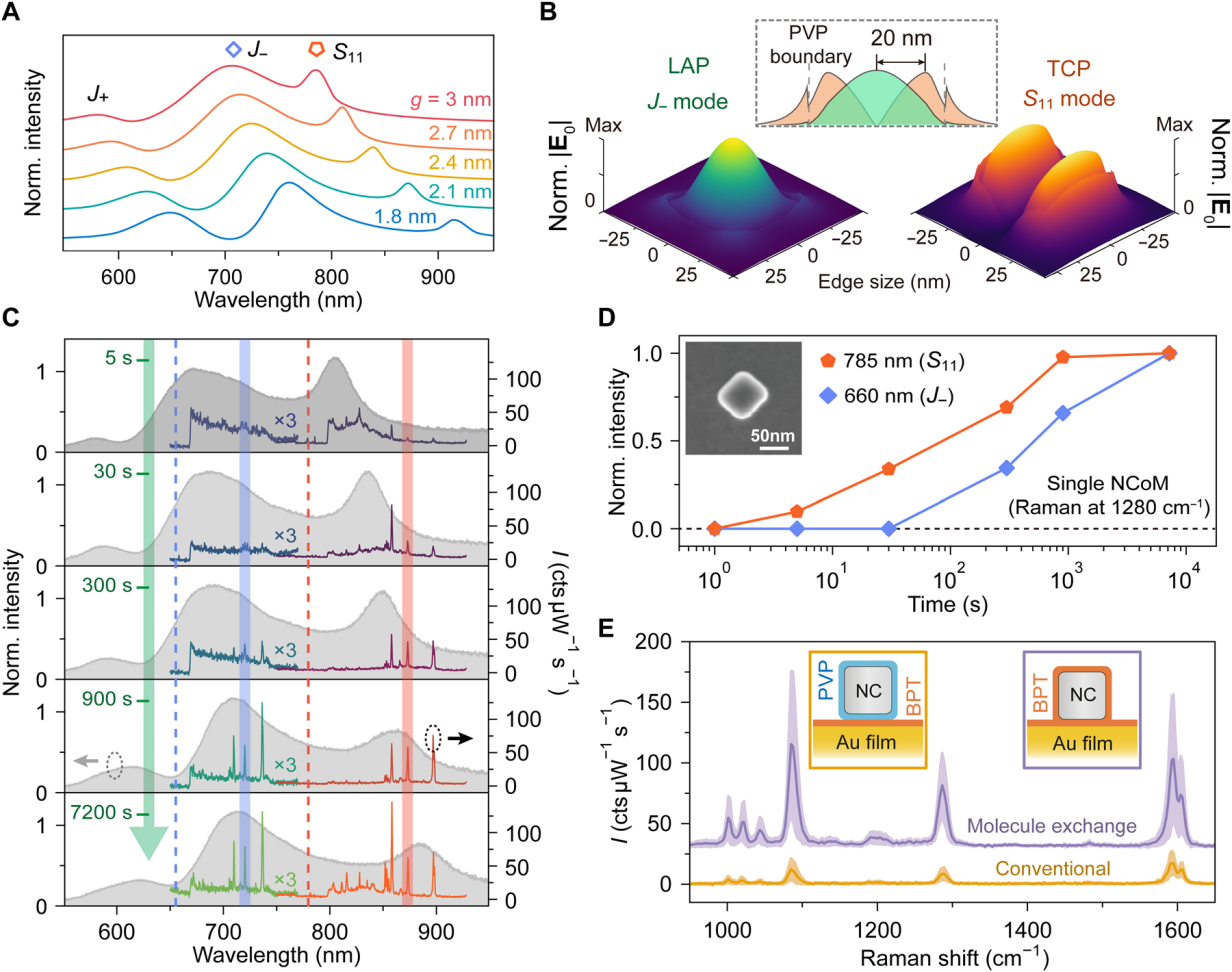
WM-SERS sensing of molecular dynamics in a single NCoM nanogap. (**A**) Simulated scattering spectra of a 70-nm-diameter Ag nanocube on a gold mirror with gap distance *g* varied from 3 to 1.8 nm, showing three plasmon modes labeled as $J_{+}$, $J_{-}$, and $S_{11}$, respectively. (**B**) 3D colormap of electric field distributions of LAP $J_{-}$ and TCP $S_{11}$ modes, respectively. The central panel shows the cross-sectional field intensity profiles of the LAP and TCP modes, illustrating their complementary spatial distributions and defining a 20-nm spatial resolving power of the WM-SERS technique. (**C**) Measured DF scattering and WM-SERS spectra (660- and 785-nm excitation, blue and orange dashed lines; cts, counts) of a single PVP-capped Ag NCoM after immersion of BPT ethanol solution with duration from 5 to 7200 s (from top to bottom), with the SEM image shown in the inset of (D). (**D**) BPT Raman peak intensity at 1280 cm^−1^ as a function of immersion time under 660- and 785-nm excitations with Raman emission at 720.8 and 872.6 nm, respectively [also labeled in (C) with blue and orange areas]. (**E**) Statistical SERS spectra from individual BPT-spaced NCoMs using conventional and our molecule exchange methods, respectively. The yellow (purple) line, area, and inset box represents the SERS mean value, error bar, and the schematics of the conventional (molecule exchange) methods, respectively.

To elucidate the temporal and spatial evolution of molecular infiltration within the nanogap, we initiated our analysis by characterizing the scattering spectra of the previously used NCoM nanostructure (Fig. 1, D and E). As shown in Fig. 2 (A and C), both electromagnetic simulations and experimental measurements revealed three distinct resonance peaks, labeled as well-documented (*55*) $J_{+}$, $J_{-}$, and $S_{11}$. Quasi-normal mode analysis (*56*) of their near-field distributions (Fig. 2B) with corresponding polarization-dependent dark-field (DF) scattering and SERS measurements (see details in fig. S8) identified the $J_{+}$ and $J_{-}$ resonances as hybridized longitudinal antenna plasmon (LAP) modes, while the $S_{11}$ mode is a transverse cavity plasmon (TCP) mode (*55*, *57*, *58*). As depicted in the field profiles in Fig. 2B (see their 2D presentations with material boundaries in fig. S6), the LAP ($J_{-}$) mode exhibits a single intensity maximum at the nanocube’s center, whereas the TCP ($S_{11}$) mode features two distinct intensity maxima near the edges, with a node at the center. The distinct spectral and spatial distributions of the $J_{-}$ and $S_{11}$ modes enable the super-resolution mapping of molecular locations within the nanogap (*52*, *53*, *59*). The ~20-nm separation between these modal maxima determines the spatial resolving power of our WM-SERS technique, enabling spatial distinction at this scale or even better. This high-contrast spatial mapping is possible because the field maximum of the LAP mode coincides with a field null of the TCP mode and vice versa. Through careful geometric tuning of the NCoM (the nanocube size and the gap thickness), we deliberately align the spectral windows of $J_{-}$ ($S_{11}$) mode with the 660-nm (785-nm) laser excitation and Stokes sideband. This spectral engineering enables us to selectively and independently address molecules at the nanogap’s center and edges, respectively, via 660- and 785-nm WM-SERS measurements.

With this spatially sensitive tool established, we applied it to track the infiltration of BPT molecules into a single PVP-capped NCoM over time after immersing the sample in solutions of BPT for controlled time intervals. Initial evidence of molecular exchange of this NCoM is provided by DF scattering spectroscopy (Fig. 2C), whose scanning electron microscopy (SEM) image is shown in the inset of Fig. 2D. After sequential immersion steps, the plasmon resonances exhibit a progressive redshift, which provides a direct optical readout of a nanoscale physical change: the subsequent replacement of the thicker PVP layer (~2 nm) with a thinner layer of BPT (~1 nm), a result that is in agreement with simulations (Fig. 2A). While this measurement confirms that exchange is occurring across the nanogap, the spatially resolved evidence for the infiltration pathway is unlocked by the time-resolved WM-SERS spectroscopy from a single NCoM (Fig. 2C). Figure 2D shows the temporal evolution of a BPT Raman band at 1280 cm^−1^ as a function of infiltration time from the single NCoM, appearing at 720.8 and 872.6 nm under 660- and 785-nm excitation, respectively (see more examples in fig. S7). The SERS signal from the edge-sensitive TCP mode (785-nm excitation) rises substantially earlier and saturates faster than the signal from the center-sensitive LAP mode (660-nm excitation). This distinct temporal lag is the direct signature of molecular dynamics, representing the finite time required for BPT molecules to infiltrate the nanochannel from the entry points at the edge to the central region. Our observation provides unambiguous, spatially resolved evidence for a centripetal infiltration pathway, verifying that molecules enter from the edges and progressively saturate the nanochannel toward its geometric center.

Beyond tracking the infiltration dynamics, we also evaluated the final outcome of this process by performing a statistical 785-nm SERS analysis on NCoMs after complete, saturated PVP-to-BPT exchange (Fig. 2E, purple line). As a benchmark, we compared these results to control samples fabricated using the conventional method, where a BPT SAM is formed on the Au film before nanoparticle deposition (Fig. 2E, yellow line, and see more discussion in fig. S4) (*12*, *16*, *60*). The comparison reveals a remarkable advantage of our strategy (see more results in fig. S5). The in situ molecule exchange approach consistently yields higher SERS enhancement. This superior performance is attributed to the formation of a cleaner and narrower nanogap. In the conventional scheme, residual ligands from the nanoparticle can become trapped upon the preformed SAM, creating an undesirably larger gap. In contrast, our exchange method effectively purges the initial spacer, which results in stronger and more reproducible field localization. While some molecules also adsorb onto the top nanoparticle surface, their SERS contribution is negligible compared to that from the extreme field confinement within the nanogap.

### Quantitative study on evolution dynamics of molecular exchange in plasmonic nanogaps

**Fig. 3.**
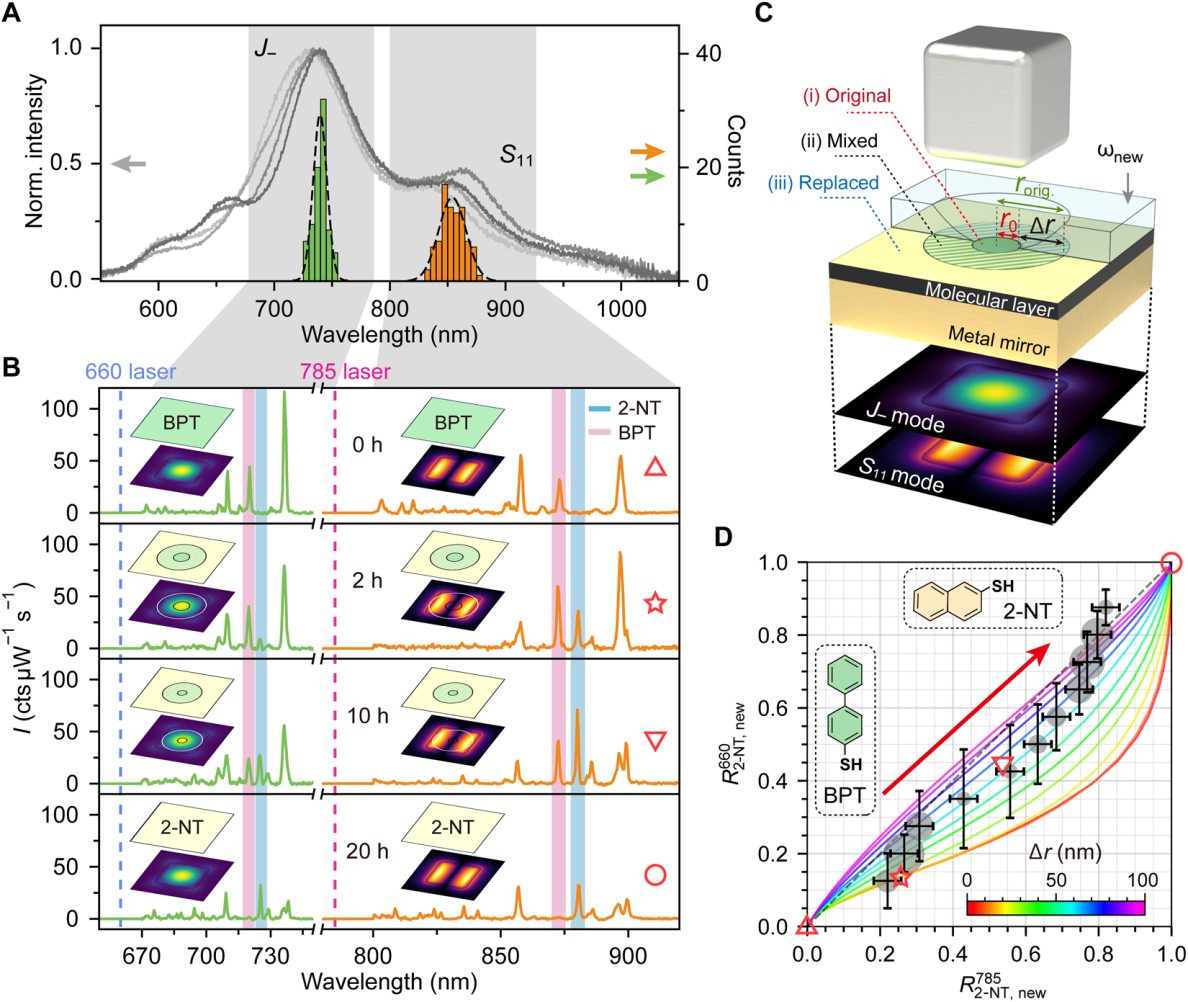
Molecular exchange mechanism in plasmonic quasi-2D nanochannels resolved by WM-SERS spectroscopy. (**A**) Representative DF scattering spectra of NCoMs with BPT and/or 2-NT spacer, along with the statistical distributions of resonance wavelengths for the LAP ($J_{-}$) and TCP ($S_{11}$) modes from 84 NCoMs. The gray area covers the spectral windows for 660- and 785-nm SERS shown in (B). (**B**) Representative WM-SERS spectra measured on individual NCoMs, representing four typical replacement stages from BPT to 2-NT molecules, as labeled in (D). The insets illustrate the molecular spatial distribution (top plane; green, BPT; yellow, 2-NT) within the nanogap mapped onto the near-field distributions of LAP and TCP modes (bottom planes). Red and blue shaded areas highlight the characteristic Raman peaks of BPT (1280 cm^−1^) and 2-NT (1380 cm^−1^). h, hours. (**C**) 3D schematics of the model for molecular exchange within a molecular-spaced NCoM nanochannel. The 3D area on top of the molecular layer represents the fractional occupancy of new molecules $\omega_{\text{new}}$ (ranging from 0 to 1). The projections of $\omega_{\text{new}}$ onto the molecular layer are colored green, green-yellow striped, and yellow, respectively, representing (i) original, (ii) mixed, and (iii) replaced zones. The corresponding WM-SERS from these three zones are mapped from the near-field distributions of the LAP and TCP modes (illustrated beneath the metal film). (**D**) Correlation plot of $R_{\text{2-NT}}^{785}$ versus $R_{\text{2-NT}}^{660}$ derived from 820 individual NCoMs, where the area of the gray circles is proportional to the nanocube numbers. Colored curves show the theoretically predicted relationship between $R_{\text{new}}^{785}$ and $R_{\text{new}}^{660}$ for varied Δr of the mixed zone.

While single-particle studies provide insight into molecular exchange within nanogaps, ensemble statistics could reveal a quantitative relationship in the dynamics. To this end, we first immersed PVP-capped NCoMs in BPT solutions until complete replacement, yielding BPT-capped NCoMs. Next, we induced controlled replacement of BPT spacers by immersing the structures in 2-naphthalenethiol (2-NT) solutions for varied durations. This results in cofunctionalized NCoMs with BPT and 2-NT coexisting in the nanogap with different proportions and spatial distributions (see Materials and Methods for details). We measured over 80 NCoMs for statistical analysis, with representative DF scattering spectra, and resonance wavelength statistics of $J_{-}$ mode and $S_{11}$ mode summarized in Fig. 3A. Both peak distribution statistics were fitted with Gaussian functions. The $J_{-}$ mode exhibits a central peak position at 740 nm with an SD σ=5.2 nm, while the $S_{11}$ mode shows a central peak at 854 nm with σ=11.8 nm. The spectral resonance alignment with the results in Fig. 2A shows sufficient structural homogeneity for reliable kinetic analysis.

Figure 3B presents the WM-SERS spectra of four representative frames of a single NCoM after different 2-NT immersion times (0, 2, 10, and 20 hours). Insets show schematics of the molecular distributions (green, BPT; yellow, 2-NT) overlaid with the $J_{-}$ and $S_{11}$ mode profiles. At 0 hours, the nanogap is fully occupied by BPT, evidenced by its characteristic Raman peak (pink shade) at 1280 cm^−1^, while the 2-NT peak at 1380 cm^−1^ is absent. After 2 hours of immersion, 2-NT begins infiltrating from the edges and partially replaces BPT, leading to coexistence within overlapping regions (further validated in Fig. 3D). In the WM-SERS spectra, the 2-NT Raman peak at 1380 cm^−1^ excited at 785 nm ($S_{11}$ mode) rises markedly, whereas the corresponding peak under 660-nm excitation ($J_{-}$ mode) shows only a slight increase. With longer immersion (10 and 20 hours), BPT is progressively replaced, and at 20 hours, its Raman signals vanish completely, leaving only 2-NT. Notably, even after 10 hours, despite 2-NT reaching the nanogap center, the BPT peak under the $S_{11}$ mode persists, indicating that residual BPT molecules remain near the edges and require longer times (20 hours) for full replacement. This suggests that the actual replacement dynamics are more complex than the simplified assumption of symmetric edge infiltration, potentially involving preferred infiltration pathways that are currently nontrivial to resolve. Since Raman peak intensities at different excitation wavelengths reflect the relative molecular locations, their intensity ratios can be leveraged to probe molecular dynamics in the quasi-2D nanochannel. To achieve this, we established a physical model and validate it through statistical analysis of experimental data (Fig. 3, C and D).

### A model for resolving nanogap molecular dynamics with WM-SERS

We define the characteristic SERS peak intensities $I_{s}^{\lambda }$, where s∈ {2‐NT, BPT} denotes the molecular species (2-NT or BPT) and λ∈{660,785} nm specifies the excitation wavelength. These correspond to $J_{-}$ mode signals (λ=660 nm) originating from nanogap center and $S_{11}$ mode signals (λ=785 nm) from the edge regions.

The absolute SERS intensity varies substantially between individual NCoMs due to subtle differences in gap size and surface morphology (*57*, *58*, *61*). To isolate the statistical features of molecular exchange from these interparticle variations, we analyze the fractional SERS contribution of the 2-NT molecules. This is calculated as the ratio of the 2-NT intensity to the total SERS intensity from both molecules, rather than using the absolute SERS intensity of 2-NT alone

$$R_{\text{2-NT}}^{\lambda }=\frac{I_{\text{2-NT}}^{\lambda }}{I_{\text{2-NT}}^{\lambda }+\eta I_{\text{BPT}}^{\lambda }},\;\forall \lambda \in \left\{660,785\right\}$$ (1)

where $\eta =\sigma_{\text{BPT}}/\sigma_{\text{2-NT}}$ is the ratio of the Raman cross sections of BPT to 2-NT in the nanogap, which is estimated to be 1.5 (see details in the Supplementary Materials and fig. S14). These ratios quantify 2-NT coverage in the central ($R_{\text{2-NT}}^{660}$) and edge ($R_{\text{2-NT}}^{785}$) regions, where $R_{\text{2-NT}}^{\lambda }=0$ denotes the pristine BPT coverage and $R_{\text{2-NT}}^{\lambda }=1$ indicates the complete 2-NT replacement. As indicated by the guide-to-eyes red arrow, the trajectories in Fig. 3D from bottom left (BPT-dominated) to top right (2-NT–saturated) can represent the spatiotemporally resolved molecular exchange progression. The different trajectories arise from the corrections to the initial assumptions by allowing for partial molecular overlap between BPT and 2-NT.

As illustrated in Fig. 3C, without loss of generality, we propose a simplified kinetic model in which an external new molecule (2-NT here) infiltration proceeds toward the center, forming three concentric zones:

1) An inner circular zone of radius $r_{0}$, termed “original” ($r<r_{0}$), containing only the original molecular spacer (BPT here, green area), where *r* denotes the distance from the nanogap center.

2) An annular transition zone ($r_{0}<r<r_{\text{orig}.}$) of width Δr, termed “mixed,” where molecules coexist (green-yellow region). The occupancy of the new molecule is described by an exponentially decaying function $\omega_{\text{new}}\left(r,r_{\text{orig}.},\Delta r\right)$, and that of the original molecule is described by $\omega_{\text{orig}.}\left(r,r_{\text{orig}.},\Delta r\right)=1-\omega_{\text{new}}\left(r,r_{\text{orig}.},\Delta r\right)$, where $r_{\text{orig}.}=r_{0}+\Delta r$ is defined as the radius of the original molecule region (see details in fig. S16).

3) An outer zone, termed “replaced” ($r>r_{\text{orig}.}$), fully occupied by the new molecule at saturation (2-NT here, yellow area).

On the basis of well-known classical electromagnetic “$E^{4}$” theory of SERS (*62*), the scattering intensity $I_{s}^{\lambda }\left(r_{\text{orig}.},\Delta r\right)$ for molecular species *s* (where s∈{new,orig.}) under excitation wavelength λ scales as follows

$$I_{s}^{\lambda }\left(r_{\text{orig}.},\Delta r\right)\propto {\left[\iint_{A}|E_{\lambda }\left(r\right)|\cdot \omega_{s}(r,\Delta r)\;d^{2}r\right]}^{2}\cdot {\left[\iint_{A}|E_{\lambda_{\text{em}}}\left(r\right)|\cdot \omega_{s}(r,\Delta r)\;d^{2}r\right]}^{2}$$ (2)

where $E_{\lambda }\left(r\right)\;\text{and}\;E_{\lambda_{\text{em}}}\left(r\right)$ denote the near-field distribution of the NCoM at wavelength λ and λ_em_, respectively. λ_em_ denotes the Raman emission wavelength by λ-nm excitation, with λ_em_ = 723 nm for λ = 660 nm and λ_em_ = 875 nm for λ = 785 nm (see details in fig. S15). “*A*” is an integration area with a radius set to 100 nm to cover the whole near-field distribution. In comparison with $R_{\text{2-NT}}^{\lambda }$, the theoretical normalized SERS intensity ratios of the new molecules are thus obtained as follows

$$R_{\text{new}}^{\lambda }\left(r_{\text{orig}.},\Delta r\right)=\frac{I_{\text{new}}^{\lambda }}{I_{\text{new}}^{\lambda }+\eta I_{\text{orig}.}^{\lambda }},\;\lambda \in \left\{660,785\right\}$$ (3)

Solid colored lines in Fig. 3D represent the computed function between $R_{\text{new}}^{660}\left(r_{\text{orig}.},\Delta r\right)$ and $R_{\text{new}}^{785}\left(r_{\text{orig}.},\Delta r\right)$, where $r_{\text{orig}.}$ spanned from 0 to 100 to cover the overall molecular exchange process. As shown in the different curves in Fig. 3D, the process are also dependent on the degrees of molecular overlap Δr. The Raman intensities of time-resolved data from the four frames of a single NCoM in Fig. 3B are calculated and overlaid in Fig. 3D as red open markers. Their positions (from left to right on the map) reflect the logical time sequence from 0 to 20 hours, indicating that the 2D map in Fig. 3D captures both the temporal and spatial distributions of the molecules.

The parameter Δr quantifies the spatial extent of molecular mixing near the interaction frontier. At the theoretical limit, Δr→∞, the model describes a complete lack of spatial confinement or directional infiltration. This scenario implies that the exchange is not limited by infiltration from the edge inwards; rather, it occurs uniformly and simultaneously across the entire nanogap area. This scenario is physically equivalent to the process of exchange occurring on an open gold surface (*44*, *54*), where different types of molecules are homogeneously mixed without anisotropic selectivity and direction of interaction caused by the geometric confinement. This stands in stark contrast to the Δr=0 case, which represents a perfectly sharp, inward-propagating replacement front without coexistence of two types of molecules.

We measured a total of 830 nanoparticles and extracted the characteristic peaks of 2-NT (1380 cm^−1^) and BPT (1280 cm^−1^) from each particle. On the basis of the ratio of their peak intensities, the nanoparticles were classified into 11 groups. The size of the gray disk represents the number of samples in each group, while the position and error bar indicate the mean and variation, respectively. The “centers of mass” of generally binned data remain well within the region predicted by the analytical solutions. By comparing the experimental data with our analytical solution, we find that the transition zone width, Δr, spans a range from 0 to 100 nm, with an average value of ~50 nm. This notable average value, which exceeds half the edge length of the 70-nm nanocubes, leads to a crucial insight: For a substantial fraction of NCoMs, the purely original central region [zone (i) in Fig. 3C] virtually does not exist throughout the process. This implies that infiltrating molecules can penetrate to the geometric center of the nanogap from the earliest stages of the replacement. It occurs even in thiol-based, SAM-spaced nanogaps (~1.3 nm) comparable to the molecular dimensions (BPT versus 2-NT), challenging the intuitive assumption that the nanoparticle would physically block molecular infiltration and seal the central region.

Furthermore, note that a subset of the experimental data and errors deviates from the theoretical range predicted by our centrosymmetric model (e.g., the top-right region of Fig. 3D). We attribute this deviation to the stochastic nature of molecular dynamics within these confined nanocube geometries. In these small-area limits, the infiltration process may not strictly adhere to the idealized symmetric rule. Instead, asymmetric infiltration—where one side of the SAM is replaced preferentially before the other—may occur, potentially influenced by the specific orientation of molecules within the SAM or local roughness of the nanogap (*61*). To test this hypothesis, we modeled a corresponding asymmetric infiltration model (see fig. S17). We found that the data points deviating from the symmetric prediction show good agreement with this asymmetric model. This suggests that the actual experimental landscape is likely a superposition of both symmetric and asymmetric infiltration pathways.

### Mapping molecular infiltration dynamics in extreme–aspect ratio nanochannels

**Fig. 4.**
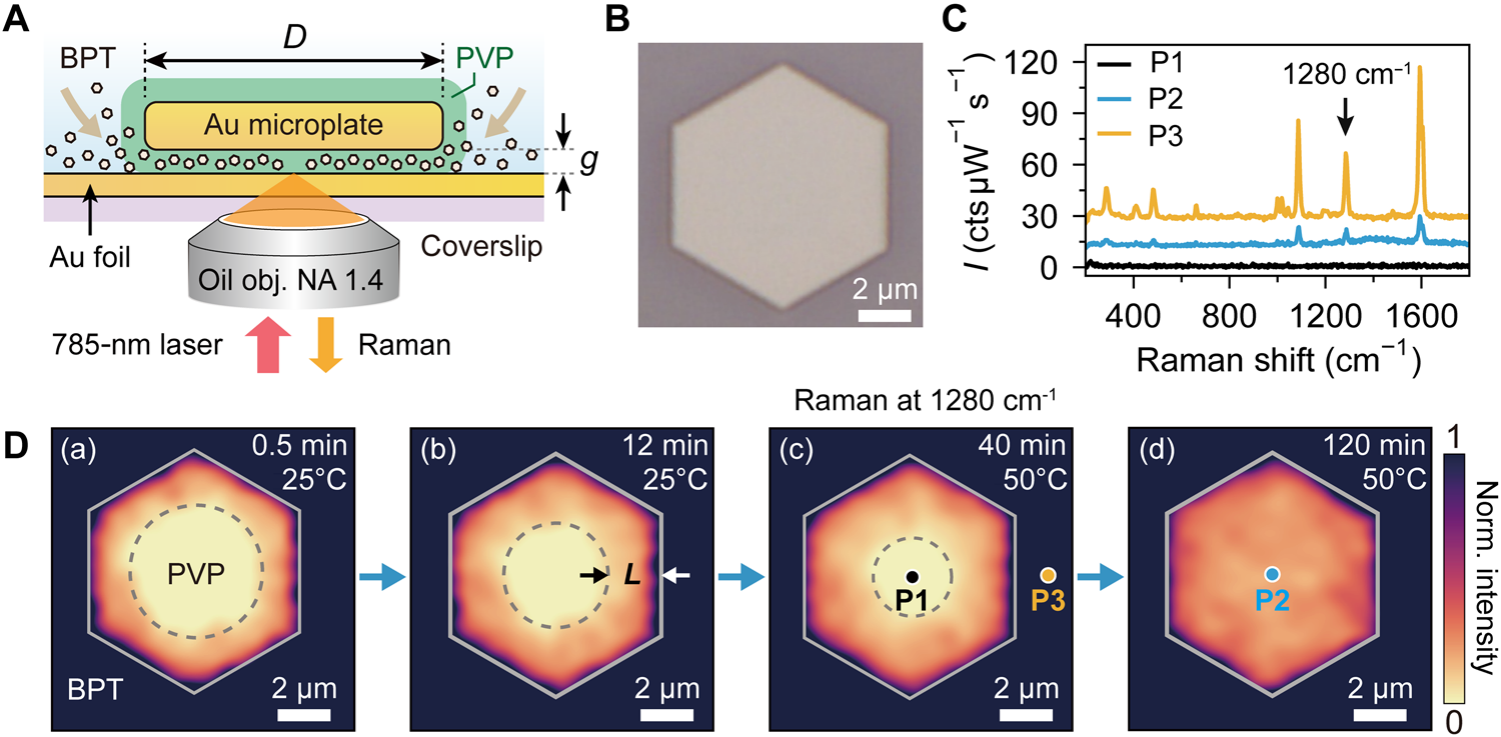
Micrometer-scale molecular infiltration and exchange dynamics. (**A**) Cross-sectional schematic of the molecular infiltration experiment in an MPoF nanochannel with microlength. The sample consists of a PVP-capped microplate (diameter *D* = 10 μm) placed on a 10-nm-thick Au foil, forming a ~ 2-nm-thick gap. The MPoF was then immersed in BPT solution for varying durations to probe molecular exchange process via SERS mapping. (**B**) Bright-field image of a representative MPoF structure. (**C**) SERS spectra of the MPoF collected at the position before [P1, black dot in (D) (c)] and after [P2, blue dot in (D) (d)] BPT replacement, respectively, compared with that at the BPT-adsorbed Au foil region [P3, yellow dot in (D) (c)]. For clarity, the blue and yellow spectra are vertically offset. (**D**) Raman intensity maps at 1280 cm^−1^ (785-nm excitation) for the MPoF in (B) after immersion in BPT solution for (a) 0.5 min at 25°C, (b) 12 min at 25°C, (c) 40 min at 50°C, and (d) 120 min at 50°C, respectively. The dashed black circle and gray hexagons indicate the original PVP-spacer region and boundary of the microplate, respectively. *L* denotes the length of the microplate region where molecular infiltration has occurred.

Building on our nanoscale model of molecular dynamics, we next sought to directly visualize this process in real space. To this end, we scaled up the NPoM-like architecture to the micrometer level by engineering nanochannels with extreme aspect ratios through an MPoF architecture (Fig. 4A). This configuration was realized by depositing a PVP-capped gold microplate (diameters, 5 to 50 μm; thickness, 50 to 100 nm) onto a 10-nm-thick rough Au foil thermally evaporated on a glass coverslip (see details in Materials and Methods). The MPoF platform generates micrometer-long plasmonic nanocavities with a gap around 2 nm (Fig. 4A). The bright-field image of the microplate was shown in Fig. 4B.

We chose a 10-nm-thick Au foil because it both transmits light and forms a quasi-uniform nanogap as plasmonic hotspots (*63*), enabling spatially resolved SERS of molecular dynamics inside the nanochannel (Fig. 4A). We investigated infiltration kinetics by immersing PVP-capped MPoF specimens in BPT solution while modulating incubation time and temperature (to accelerate the exchange). Time-dependent Raman mapping across entire microplates revealed the spectral and spatiotemporal progression of BPT replacement of the PVP spacer layer. Representative spectra and spatial maps of the Raman band at 1280 cm^−1^ are presented in Fig. 4 (C and D), respectively.

Molecular infiltration and exchange are directly visualized via mapping of BPT’s characteristic SERS peak in Fig. 4D. The infiltration patterns [0.5- to 40-min incubation; Fig. 4D (a to c)] reveal three distinct and progressively evolving zones: (i) uniform SERS signals outside the microplate boundary (its representative spectrum shown as yellow line in Fig. 4C, position P3), corresponding to BPT monolayers adsorbed on the foil’s plasmonic hotspots induced by its roughness; (ii) a central signal-deficient circular region (black line in Fig. 4C, position P1), indicating unmodified PVP spacers; and (iii) a region near the microplate edge showing weaker but discernible BPT signatures, confirming nanochannel infiltration over distances of *L* ~ 2 μm as shown in Fig. 4D (b). With increasing incubation time, the central PVP region progressively disappears [Fig. 4D (d)], while BPT signals gradually emerge at the nanogap center [shown by the comparison of spectra in Fig. 4C at the positions P1 and P2 in Fig. 4D (c and d)]. These provide a direct visualization of molecular propagation and exchange.

This real-space molecular Raman mapping performed on the ~10-μm-long, ~2-nm-thick 2D channel validates our predictive model shown in the last sections. Critically, we demonstrate unprecedentedly long molecular infiltration lengths in extreme confinement environments (~10^3^ aspect ratio). Our findings are particularly important, as molecular dynamics within these extreme–aspect ratio 2D nanochannels—where confinement is comparable to the molecular scale—are expected to differ fundamentally from behavior in larger spaces. While other platforms such as plasmonic nanopores exist, their channel dimensions are often substantially larger than the target molecules, leading to stochastic measurement challenges (*3*). In contrast, our system provides a versatile platform for simultaneous optical characterization under these extreme, molecular-scale confinement conditions.

### Ultrasensitive molecular sensing of a single plasmonic nanogap

Having established the molecular exchange mechanism within plasmonic 2D nanochannels, we now evaluate their postsensing capabilities. An effective sensing platform was prepared by firstly assembling 150-nm-diameter CTAC-capped gold nanoparticles onto gold films, followed by immersion the sample in BPT solution to achieve saturated spacer replacement.

**Fig. 5.**
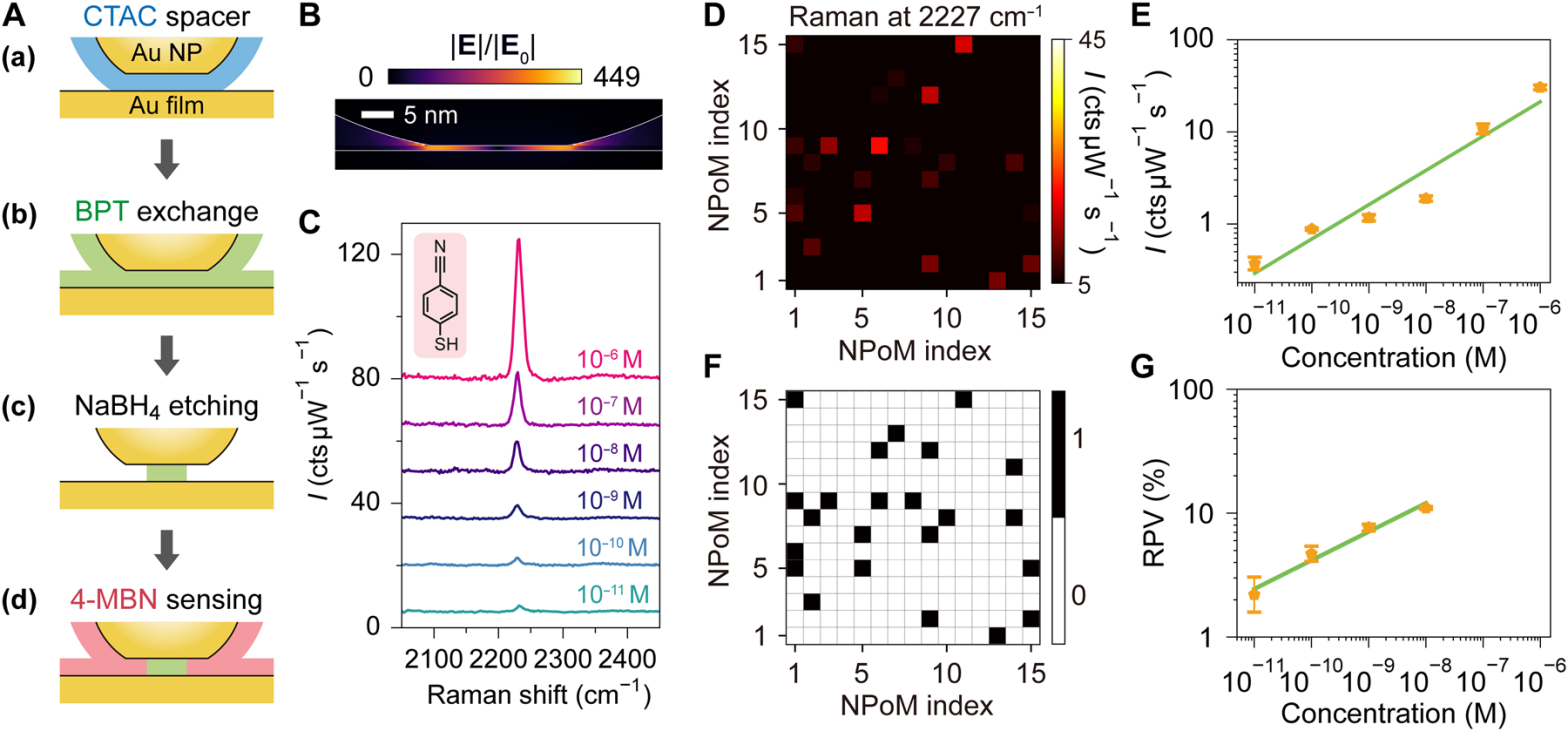
In-situ ultrasensitive molecular postsensing via a single NPoM plasmonic nanogap. (**A**) Schematic of the selective nanogap engineering process for molecular postsensing: (a) a 150-nm-diameter CTAC-capped Au nanoparticle (AuNP) deposited on a Au film to form a NPoM nanogap; (b) a BPT-spaced nanogap is formed via the molecular exchange method; (c) partial etching of edge BPT molecules using NaBH_4_ solution preserves central BPT to maintain nanochannel integrity; (d) subsequent immersion in analyte (4-MBN) solution enables analyte adsorption into the etched nanogap regions. (**B**) Near-field distribution (at 880 nm) of the 150-nm NPoM, providing field enhancement over 449 times. (**C**) Representative 785-nm SERS spectra showing the characteristic 4-MBN peak at 2227 cm^−1^ across concentrations varied from 10^−6^ to 10^−11^ M. The spectra are vertically offset for clarity. (**D**) Raman intensity map (2227 cm^−1^) of 225 individual NPoM structures at 10^−8^ M 4-MBN, arranged in a 15 × 15 grid. (**E**) Statistical SERS intensity at 2227 cm^−1^ of the NPoM as 4-MBN concentrations varied from 10^−6^ to 10^−11^ M. Green line denotes a linear fit. (**F**) Digital SERS map derived from (D) by applying an intensity threshold (see fig. S12). (**G**) Ratio of positive voxels (RPV) based on digital SERS analysis (F) versus 4-MBN concentration. The green line denotes a linear fit.

To create active binding sites for subsequent analyte detection, we treated the BPT-spaced NPoMs with NaBH_4_ solution (procedures shown in Fig. 5A and see details in Materials and Methods). This partial etching process is expected to initiate at the more accessible peripheral regions of the nanogap, creating a higher density of vacant sites near the edges for analyte infiltration. This edge region supports well-defined hotspot regions with field enhancement over 400-fold for molecular sensing (Fig. 5B). We term it postsensing because these NaBH_4_-etched open quasi-2D channels (chambers) with nanogap hotspots can serve as a general SERS substrate, capable of detecting molecules deposited afterward (see details in fig. S9).

To evaluate the sensing performance, we incubated the modified NPoMs [Fig. 5A] in 4-mercaptobenzonitrile (4-MBN) solutions of varying concentrations, allowing the analyte to occupy the nanogap regions previously etched by NaBH_4_. The representative SERS spectra are shown in Fig. 5C and fig. S11. The characteristic nitrile stretch of 4-MBN at 2227 cm^−1^ is spectrally well separated from the Raman bands of the BPT spacer, enabling unambiguous signal characterization. For a direct comparison between samples with and without NaBH_4_ treatment, we find that the SERS intensity of 4-MBN after NaBH_4_ treatment is more than 20 times stronger than that of the untreated control (see fig. S10). For each concentration (from 10^−6^ to 10^−11^ M), we measured 225 individual NPoMs (see details in fig. S13). To visualize the statistical variations, we arranged the SERS intensities of the characteristic peak into a 15 × 15 grid map, as exemplified for 10^−8^ M in Fig. 5D. By averaging the intensities for each concentration, we plotted the SERS intensity as a function of molecular concentration (Fig. 5E), which displays a generally monotonic, quasi-linear trend. Recent advances in SERS show that the linear relationship between signal intensity and concentration breaks down at low concentrations; we also used a digital SERS approach (*64*, *65*). This technique more accurately describes analyte presence by quantifying the probability of discrete molecular adsorption events (“on” versus “off”). To implement this, we set an intensity threshold for the 4-MBN peak: Signals above the threshold were assigned a value of 1, and those below were assigned 0 (fig. S12). This converted the analog intensity map (Fig. 5D) into a binary map of detection events (Fig. 5F and see more results in fig. S13). Plotting the resulting ratio of positive voxels (RPV) against concentration (Fig. 5G) revealed an improved linear dynamic range. This digital analysis further confirms our ability to detect 4-MBN down to 10^−11^ M, establishing the ultrasensitive sensing of our platform for nonresonant small molecules toward single-molecule detection limits (*64*, *66*).

### Real-time molecular transport and sensing in a single plasmonic nanochannel

**Fig. 6.**
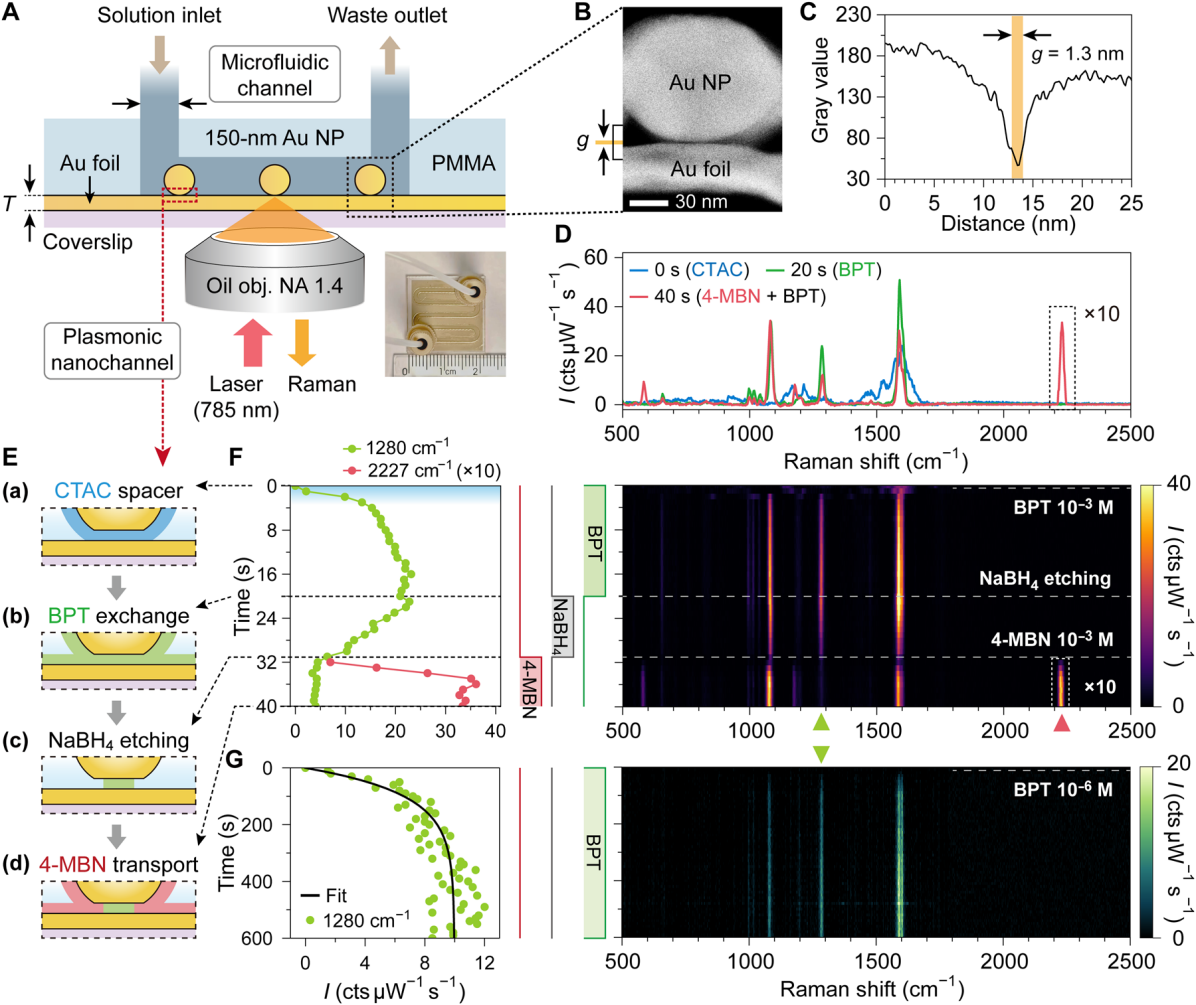
In situ real-time molecular SERS sensing via a single sub–2-nm plasmonic nanochannel. (**A**) Experimental setup integrating an NPoF assembly within a microfluidic channel. A 150-nm CTAC-capped AuNP on Au foil (thickness *T* = 10 to 35 nm) is excited by a 785-nm laser from the coverslip side. Inset: Photograph of the integrated chip. (**B** and **C**) High-resolution STEM cross section of the NPoF nanochannel with a BPT SAM spacer (B) and the corresponding grayscale intensity profile (C) showing a gap thickness *g* ≃ 1.3 nm (also see fig. S19). (B) is the is the same as fig. S19B. (**D** to **F**) Real-time monitoring of molecular transport in a single NPoF nanochannel (*T* = 35 nm). (E) Schematic sequence of the exchange process: (a) initial CTAC spacer, (b) BPT replacement, (c) partial etching by NaBH_4_, and (d) 4-MBN transport. (F) Corresponding time-resolved SERS intensity map (right) and extracted peak trajectories (left) at 1280 cm^−1^ (BPT) and 2227 cm^−1^ (4-MBN). (D) Representative SERS spectra extracted at the corresponding stages of the process. (**G**) Sensitivity test using 10^−6^ M BPT solution on a single NPoF (*T* = 10 nm). Left: Kinetic trace of the peak intensity at 1280 cm^−1^ fitted with a Langmuir model (black line). Right: Corresponding time-resolved SERS map.

Real-time molecular detection is an essential capability in Raman spectroscopic applications. We integrated a nanoparticle-on-foil (NPoF) architecture with a microfluidic device, as depicted in Fig. 6A (inset shows the integrated chip; see details in fig. S20). The NPoF platform, comprising 150-nm-diameter CTAC-capped gold nanoparticles on a thin Au foil (35 nm in thickness for the real-time continuous molecular replacement experiment in Fig. 6, B to F and 10 nm in thickness for the low-concentration experiment in Fig. 6G). This configuration is designed to facilitate both 785-nm laser excitation and in situ single-particle SERS signal collection. To structurally validate the sub–2-nm plasmonic nanochannel formed within the ligand-defined nanogap, we performed scanning transmission electron microscopy (STEM) on a focused ion beam–milled cross section (Fig. 6B and see details in the Supplementary Materials). As quantified in Fig. 6C and fig. S19, the gap thickness between the nanoparticle and the foil is ~1.3 nm (see detail analysis in the Supplementary Materials).

Transitioning from passive, immersion-based molecular kinetics (Figs. 1 to 5) to a dynamic and application-relevant format, we implemented the molecular exchange sequence within this flow-controlled microfluidic transport environment (Fig. 6). The process involves a multistep protocol where the initial CTAC ligand layer [Fig. 6E (a)] is replaced by BPT [Fig. 6E (b)], subsequently partially etched by NaBH_4_ [Fig. 6E (c)], and lastly utilized to host and sense the transport of 4-MBN [Fig. 6E (d)]. By continuously monitoring specific Raman fingerprints, we tracked the entire evolution of these molecular events in real time. The real-time spectral evolution (Fig. 6F) reveals distinct kinetic stages. Initially, the characteristic BPT fingerprint at 1280 cm^−1^ rises sharply within the first 4 s and reaches saturation by ~20 s, marking the near completion of ligand replacement. Upon the subsequent infusion of NaBH_4_, the BPT layer undergoes gradual etching. Crucially, we controlled this etching to intentionally retain a fraction of the BPT molecules; this residual layer serves as a spacer to support the nanogap and prevent the collapse of the plasmonic nanochannel, evidenced by the persistent signal at 1280 cm^−1^ at 32 to 40 s. Following the injection of 4-MBN, we observed rapid transport into the nanochannel, with the mode at 2227 cm^−1^ saturating within a remarkably short window of 8 s. The corresponding spectral map (Fig. 6F, right) visualizes this transition, distinctly showing the decay of BPT signatures, followed by the emergence of the 4-MBN peak. Note that the Raman intensity at 2227 cm^−1^ of 4-MBN is magnified by a factor of 10 for visual clarity.

To further validate the platform’s sensitivity and kinetic behavior, we perfused a 10^−6^ M BPT solution through the microfluidic channel while performing continuous in situ SERS on a single NPoF (Fig. 6G). The temporal evolution of the peak intensity at 1280 cm^−1^ follows a clear exponential trajectory that aligns consistently with the Langmuir kinetic model for molecular infiltration and saturation (*54*) (see the Supplementary Materials). These results not only validate our analysis of the molecular dynamics but also demonstrate the efficacy of NPoM-like systems as versatile, all-in-one transport-and-probe platforms capable of resolving interactions within nanoconfined 2D channels.

## DISCUSSION

Our work fundamentally unlocks a postsensing paradigm for bottom-up plasmonic nanogap architectures. While bottom-up fabrication has been superior to top-down methods in creating reliable, subnanometer gaps with exceptional field confinement, its potential has been constrained by the perceived “closed” nature of the nanojunctions. By demonstrating that NPoM-like gaps function as open nanochannels accessible via molecular exchange and transport, we bridge the gap between high-performance plasmonics and in situ dynamic analysis. While our work focused on individual NPoMs to elucidate the underlying mechanism, this open-channel functionality inherently benefits from the well-known scalability of the NPoM platform, which offers near 100% yield over macroscopic areas at a fraction of the cost of top-down methods (*11*, *12*).

Our experimental validation intentionally focused on the simplest ligand-spaced NPoM configuration to reveal its counterintuitive (*13*) yet powerful capabilities. This basic architecture offers a dynamic nanochannel where initial weak-binding ligands can be readily replaced by most small molecules. However, even when a dense SAM formed by thiols occupies the gap, the channel remains accessible. Subsequent exchange with molecules possessing smaller binding energy is not prevented but simply becomes less kinetically favorable. For example, our results show that MB can still infiltrate the gap to replace the preadsorbed BPT monolayer (see fig. S18 for details). This finding challenges the notion of a sealed channel after strong ligand adsorption and reinforces the high versatility of our platform.

Furthermore, we measured the scattering spectra before and after molecular exchange (see fig. S21 for details) and monitored the nanoparticle motion under both static and dynamic (microfluidic) conditions (see movies S1 and S2). These measurements show that the vast majority of nanoparticles remains immobilized on the Au film, exhibiting no noticeable displacement or rolling. The observed molecular infiltration and exchange, therefore, are believed to occur within the nanogap rather than through nanoparticle motion.

On the other hand, the partial etching strategy demonstrated in Fig. 5A represents a straightforward solution, creating active sensing sites while preserving the structural integrity of the nanogap. This simple, low-cost approach is sufficient and ideal for many applications, where, e.g., the postadsorption of analytes is ideal for differential measurements by tracking weak signal responses (*29*, *38*, *39*). In chiroptical sensing (*67*), this would allow the intrinsic chiral response of the plasmonic structure to be measured first, followed by the introduction of chiral molecules, effectively decoupling the analyte’s signal from the system’s background chirality. This concept extends to tracking chemical reactions and energy transfer (*19*, *23*–*25*, *68*) or to any externally stimulated transformation that is sensitive to the plasmonic extreme local field. In studies of photocatalysis that prefer a well-defined metallic surface (*69*), the molecular exchange process we demonstrated could be an intrinsic and necessary step to remove the original ligands that would otherwise impede the reaction.

Looking ahead, the NPoM all-in-one transport-and-probe platform offers various possibilities, from accessible, cost-effective solutions to highly engineered, high-performance systems. For applications demanding greater specification and reproducibility, the simple ligand spacer could be replaced with more sophisticated scaffolds such as MOFs (*40*), host-guest complexes (*20*), artificial channels (*2*), or using nanoparticles with concave surfaces (*70*). This path, however, presents a trade-off between performance and complexity, requiring careful consideration based on the specific application. For more ambitious goals, such as achieving fast mass transport, the platform must be engineered to combine the performance metrics of plasmonic nanopores (*3*, *4*) with the geometric advantages of the 2D nanochannel—namely, extended analyte dwell times and the optimal field enhancement of the NPoM nanogaps. The future development of this platform can draw inspiration from the capabilities established in these related fields, aiming to track the transport and reactions of single molecule with ever-higher spatiotemporal resolution. This could be achieved by integrating advanced super-resolution techniques (*71*) in terms of spatial localization (*72*, *73*) and molecular orientation (*74*), which would complement the WM-SERS method used here. Furthermore, analogous to established strategies for dynamic control in the broader field of 2D nanochannels (*8*, *10*), active tuning of the NPoM gap size could enable molecular sieving, adding a layer of selectivity to the ultrasensitive detection. Last, the microscopic details of the surface chemistry in such a nanochannel—including molecular orientation within the gap and preferential binding sites—warrant further investigation to build a complete picture of this complex nanoscale environment.

In conclusion, this study establishes molecular exchange as a transformative methodology that redefines ligand-capped plasmonic nanocavities as open-channel, all-in-one transport-and-probe platforms. Our approach preserves the defining advantages of bottom-up assembly with subnanometer precision and reproducible electromagnetic hotspots, while overcoming the limitation of analyte inaccessibility. We have demonstrated that this architecture enables postfabrication molecular infiltration using the super-resolved WM-SERS technique, achieving near-single-molecule sensitivity (10^−11^ M) for nonresonant analytes and real-time monitoring in microfluidic environments. This approach, leveraging powerful field localization to probe behavior in ultraconfined geometries, represents a valuable methodology at the interface of nanoscale physics, nanophotonics, and molecular sensing. By visualizing molecular infiltration over unprecedented length scales (>5 μm) within tightly confined (~2 nm) channels with extreme aspect ratios (>10^3^), we resolve important questions regarding molecular ingress in NPoM-like ultrasmall nanogaps. The proposed centripetal replacement model, which challenges classical infiltration expectations, provides a useful framework for understanding these processes. Fundamentally, this work establishes open-channel NPoM-like structures as a simple, scalable, and universal architecture for advancing in situ ultrasensitive science and exploring intriguing transport phenomena at the nanoscale.

## MATERIALS AND METHODS

### Numerical simulations

Simulations were performed with the finite element method package COMSOL Multiphysics 6.2. Two types of computation were implemented: (a) For the model profiles of $J_{-}$ and $S_{11}$ modes, we used quasi-normal mode analysis (*56*), and (b) for the field enhancement at the excitation and emission wavelengths, we used a p-polarized oblique incident plane wave to simulate experimental conditions. For both simulations, perfectly matched layers were used as boundary conditions to absorb the propagating waves in the free space. The permittivity of gold and silver was taken from Johnson and Christy (*75*) and Olmon et al. (*76*), respectively. The refractive index of PVP was taken as 1.5, while that of BPT was 1.4.

### Metal film fabrication

Four types of metal films were prepared: The 100-nm Au/Cr film was fabricated by sequentially depositing 1-nm chromium and 100-nm gold onto a silicon substrate. Similarly, the 10-nm Au foil were prepared by depositing the corresponding metal layers onto a glass coverslip. The template-stripped Au film (*39*) was prepared by thermally evaporating Au layer onto a clean silicon wafer. One consists of a 150-nm-thick layer bonded to 12 mm–by–12 mm glass coverslips, and the other consists of a 35-nm-thick layer bonded to 22 mm–by–22 mm glass coverslips. The bonding process utilized an ultraviolet-curable optical adhesive (NOA61). After ultraviolet curing, the silicon wafer was separated from the gold film using a razor blade, yielding template-stripped gold surfaces with high smoothness for both film thicknesses.

### Types of nanoparticles

Nanoparticles with different ligands were purchased from MeLab platform, including 70-nm Ag nanocubes capped with CTAC and PVP ligands, 80-nm Au nanoparticles (AuNPs) capped with CTAC and citrate, as well as 100- and 150-nm-diameter AuNPs, both capped with CTAC ligands.

### Molecular solution

A series of molecules were used as interfacial spacer layers in sample fabrication: BPT, 4-MBN, BSe, and MB. All molecules were purchased from Aladdin Chemistry Co. Ltd. NaBH_4_ solution was chosen to remove molecular adsorbates from the gold surface (*41*). Specifically, NaBH_4_ (Sinopharm Chemical Reagent) was dissolved in a water/ethanol mixed solvent (1:1 in volume). To achieve complete removal of the molecules within the nanocavities, a solution containing 0.2 g of NaBH_4_ in 40 ml of solvent was prepared. In contrast, for partial removal and the microfluidic experiments, a dilute solution consisting of 0.01 g of NaBH_4_ in 40 ml of the solvent was used. The samples were then immersed in this solution for different durations to achieve varying cleaning effects, rinsed with ethanol, and lastly dried with nitrogen flow. All molecules were of analytical grade and were used without further purification.

### Fabrication of nanocavity structures by two methods

The nanocavity structures were fabricated using two distinct approaches: the conventional method and the molecule exchange method. In the conventional approach, a SAM was first formed on the Au film, followed by the deposition of metal nanoparticles. Conversely, the molecule exchange method involved the initial deposition of nanoparticles onto the Au film to establish the nanocavity architecture, followed by immersion in the target molecular solution to induce exchange (as illustrated in fig. S4). For the nanoparticle deposition step in both methods, 20 μl of the nanoparticle suspension (0.01 mg/ml) was applied onto the substrate. Following a static incubation period to facilitate particle adsorption, the excess suspension was carefully removed, and the substrate was dried under a gentle stream of nitrogen gas (N_2_). A summary of the 11 prepared samples (labeled A to K) is provided in table S1.

### Multiple molecular exchange in a single nanocavity

The 70-nm Ag cubes capped with PVP were first deposited on a template-stripped Au film. The samples were subsequently characterized by Raman spectroscopy. Subsequently, the samples were immersed in 1 mM MB aqueous solution for 5 s and 2 hours, in 1 mM BSe ethanol solution at 60°C for 5 min and 2 hours, and in 1 mM BPT ethanol solution for 2 min and 2 hours, respectively. After each immersion, the samples were rinsed with ethanol, dried with nitrogen flow, and then characterized by Raman spectroscopy. The experimental results are shown in Fig. 1D.

### Molecular exchange within micrometer-long nanocavities

PVP-capped Au microplates (diameter, ~10 μm) were deposited on 10-nm-thick Au foil. At room temperature, the samples were sequentially immersed in 1 × 10^−3^ M BPT solution for 30 s and 12 min, followed by immersed in the same solution at 50°C for 40 min and 2 hours. After each immersion step, the samples were rinsed with ethanol, dried with nitrogen flow, and then characterized by Raman spectroscopy. The experimental results are shown in Fig. 4.

### Microfluidic SERS measurement

AuNPs (150 nm) with CTAC ligands were deposited on a 10-nm-thick Au foil to form nanocavity structures. A microfluidic template is composed of three primary components. First, 150-nm AuNPs capped with CTAC ligands were deposited onto a Au foil to construct the NPoF architecture. Subsequently, a microfluidic template with cross sections measuring 1.2 mm by 0.2 mm, was fabricated from polyethylene terephthalate (PET). A poly(methyl methacrylate) (PMMA) cover plate and a PET template were assembled with the functionalized NPoF substrate to form a “sandwich” configuration. The template is tightly attached to the film to prevent solution leakage (as shown in fig. S20). The pressure exerted by the PET is applied only to the sealing areas, while the nanoparticles of interest used for optical measurements are located within the open microfluidic channels. Therefore, the nanoparticles of interest do not come into direct contact with the PMMA template and remain intact.

Time-resolved Raman measurements in Fig. 6 were carried out using a 100× oil objective [Olympus; 1.4 numerical aperture (NA)]. For the measurements shown in Fig. 6F, the NPoM structures were assembled using 150-nm-diameter gold nanospheres on a 35-nm-thick Au foil. The solutions of BPT and 4-MBN were prepared both at a concentration of 1 × 10^−3^ M and NaBH_4_ at 6.6 × 10^−3^ M. In contrast, the samples for Fig. 6G were prepared using 150-nm-diameter gold nanospheres on a 10-nm-thick Au foil, with a reduced BPT concentration of 1 × 10^−6^ M. Throughout all microfluidic measurements, the flow rate was strictly controlled at 0.02 ml/s.

### Optical setup and data acquisition

A multifunctional optical system was constructed, and the samples were precisely positioned using a closed-loop translation stage. The experimental optical setup is illustrated in fig. S1. For bright-field imaging, white light from a halogen source was reflected into the 100× objective (Olympus; 0.9 NA) and focused onto the sample surface. The reflected signal was collected by the same objective, focused by a 200-mm focal length lens onto a complementary metal-oxide semiconductor camera (Tucsen, MIchrome 20), and recorded as bright-field images. For DF scattering spectra, a halogen lamp with DF module was used to provide annular illumination. The light was focused onto the sample through the objective, and the scattered signal was collected by the same objective, directed through a series of beam splitters (LBTEK, BS2119-C, R/T 10/90), and then focused by a 200-mm focal length lens into the spectrometer (IsoPlane 320, Princeton Instrument; charge-coupled device, BLAZE 400HRX). A grating (150 lines/mm) was used for dispersion to obtain the DF scattering spectra.

For Raman spectroscopy, continuous-wave lasers at 660 nm (CNI laser, MSL-FN-660) and 785 nm (CNI laser, MSL-III-785L) were used as excitation sources. The laser beams were directed into the objective via mirrors and focused onto the sample surface. The Raman signal was collected by the same objective, passed through a series of beam splitters, and then focused by a 200-mm focal length lens into the spectrometer with a grating (300 lines/mm). The acquisition time for Raman signals varied depending on the specific experimental conditions. The integration time was set to 10 s for Fig. 1, 5 s for Figs. 2 and 3, and 1 s for Figs. 4 and 6. For the sensitivity detection in Fig. 5, an integration time of 5 s was used for concentrations ranging from 10^−6^ to 10^−8^ M, while a longer integration time of 40 s was applied for lower concentrations (10^−9^ to 10^−11^ M) to ensure adequate signal-to-noise ratio.

### Raman peak intensity extraction

To ensure accurate quantitative analysis of the SERS kinetics, the raw Raman spectra were first preprocessed using an asymmetric least-squares algorithm to subtract the background and correct the baseline drift. Following this, the signal evolution was evaluated using two metrics: (i) peak intensity, extracted directly from the maximum height of the characteristic peaks, and (ii) integrated peak area. For the area calculation, we performed Lorentzian fitting within a spectral window of ± 35 cm^−1^ (~1.5 nm) centered at the target peak position to obtain the integrated intensity.

For example, in the microfluidic experiments (Fig. 6F), we monitored the temporal evolution of the characteristic peaks at 1280 and 2227 cm^−1^. As shown in fig. S2, the relative intensity trends derived from both methods (peak height and peak area) are nearly identical. This consistency is observed because the full width at half maximum of the Raman peaks remains narrow and highly stable throughout our experiments, with no notable broadening or shape distortion. Given this stability, using peak height provides an accurate measure of the signal while maintaining simplicity in data presentation.

## Supplementary Materials

### The PDF file includes:

Supplementary Text Figs. S1 to S21 Tables S1 and S2 Legends for movies S1 and S2 References
