# Supplementary material for: Spatiotemporal Raman probing of molecular transport in sub–2-nm plasmonic quasi-2D nanochannels
Source: Sci Adv. 2026 Feb 25;12(9):eaec3641. doi: 10.1126/sciadv.aec3641 (PMC12935047; doi:10.1126/sciadv.aec3641)
Supplement: Supplementary file 1 — Supplementary Text Figs. S1 to S21 Tables S1 and S2 Legends for movies S1 and S2 References [file sciadv.aec3641_sm.pdf]

Science Advances
AAAS

Supplementary Materials for

Spatiotemporal Raman probing of molecular transport in sub–2-nm plasmonic quasi-2D nanochannels

Haoran Liu *et al.*

Corresponding author: Huatian Hu, huatian.hu@iit.it; Wen Chen, wchen@lps.ecnu.edu.cn; Hongxing Xu, hxxu@hnas.ac.cn

Sci. Adv. **12**, eaec3641 (2026)
DOI: 10.1126/sciadv.aec3641

The PDF file includes:

Supplementary Text
Figs. S1 to S21
Tables S1 and S2
Legends for movies S1 and S2
References

Other Supplementary Material for this manuscript includes the following:

Movies S1 and S2

Comparative analysis of molecule-exchange vs. conventional fabrication for NPoMs

To directly compare the performance differences between samples prepared using the molecular exchange method and the conventional method, we constructed various types of NPoM samples based on multiple combinations of raw materials. The DF and SEM images of some samples are shown in fig. S3.

The combinations were designed based on four key variables: (1) the type of Au substrate, (2) the analyte molecule, (3) the size of the nanoparticles, and (4) the type of ligands. In total, 11 distinct samples were prepared for comparative testing (detailed compositions are provided in table S1). Each type of NPoM sample was fabricated using both the conventional bottom-up method and the molecule exchange method.

The conventional bottom-up preparation procedure is illustrated in fig. S4B. Specifically, the gold film was first immersed in a solution containing the target molecule at a defined concentration for 24 h to allow for the formation of a dense and ordered self-assembled monolayer (SAM) on the metal surface. The film was then rinsed with ethanol to remove excess molecules and dried with a nitrogen flow. Subsequently, metal nanoparticles coated with surfactants were deposited onto the molecular layer to form the NPoM structure.

In contrast, the molecule exchange method (shown in fig. S4C) begins with the deposition of surfactant-coated nanoparticles directly onto the gold substrate. The assembled sample was then immersed in a target molecule solution for 24 h, during which the analyte molecules gradually replaced the original surfactant layer. After immersion, the sample was similarly rinsed with ethanol and dried with a nitrogen flow.

For each structural configuration, samples prepared by both methods were analyzed via Raman spectroscopy, with signal intensities compared by averaging multiple spectra. Specifically, the characteristic peaks at 1280 cm^{-1} for BPT and 1073 cm^{-1} for 4-MBN were selected for the intensity analysis. The results are presented in fig. S4D, including a dashed reference line indicating a Raman intensity ratio of 1. It is evident that samples prepared using the molecular exchange method exhibited stronger Raman signals across most compositions. For example, the difference in the type G sample as shown in fig. S4E.

This performance difference is primarily attributed to the residual presence of surfactants within the nanogaps of conventionally prepared samples. Common surfactants, particularly for PVP-coated nanocubes (types C and D), typically exhibit thicknesses of ~2 nm (*39, 77*). In contrast, the molecule exchange method enables the substitution of these surfactants by target molecules (e.g., BPT forming a ~1.3 nm self-assembled monolayer), leading to substantially narrower nanogap distances and enhanced local field intensities (*14*). However, for citrate- and CTAC-capped nanoparticles, ligands are believed to rearrange or be partially displaced upon deposition, introducing an initially thinner nanogap than in solution (*78, 79*). Consequently, the relative reduction in gap distance is smaller, yielding only slight enhancement. We also note that the enhancement depends on the degree of resonance overlap. The molecular exchange induces a redshift of the plasmon resonance, which optimizes spectral alignment for certain vibrational modes, yielding stronger enhancement. For other modes or molecules, however, this shift may not improve—and may even reduce—the resonance overlap, resulting in slight enhancement.

The role of spacer molecules in enabling nanochannel accessibility

The process involves depositing CTAC-capped 80-nm-thick gold nanoparticles onto a gold film pre-functionalized with a self-assembled monolayer (SAM) of BPT molecules, as illustrated in fig. S5A. A single nanoparticle is selected for DF scattering and Raman measurements. The sample is then immersed in ethanol for 24 h to remove the CTAC ligands, followed by repeated optical measurements of the same particle. The results are shown in fig. S5B and S5C. The comparison of the Raman signals before and after ethanol treatment reveals a substantial enhancement in signal intensity following the removal of CTAC, which is attributed to the reduced nanogap distance and the resulting increase in the local electromagnetic field within the cavity. Additionally, DF scattering spectral analysis shows a pronounced red shift of the peak center after CTAC removal, confirming that the reduced gap height alters the plasmonic resonance characteristics of the cavity.

fig. S5E shows the evolution of the DF peak center as the sample is immersed in ethanol for 0.5, 1, 2, 6, 12, and 24 h. As CTAC gradually dissolves into the ethanol, the gap height decreases progressively, leading to a red shift of the DF peak center. When the gap became sufficiently narrow, quantum tunneling occurred, resulting in a gradual blue shift of the DF peak (*58, 80*). Following ethanol treatment, the sample was further immersed in a BPT solution for 24 h. At this stage, the molecular channels were no longer accessible, preventing BPT molecules from entering the gap region, and thus the Raman signal became nearly undetectable.

The molecular exchange method does not strictly require that the target molecule exhibit substantially stronger binding affinity to the metal surface compared to the initial ligand (molecular channel). In this experiment, two sample preparation strategies were employed to construct initial nanocavities, with BPT molecules serving as the channel species. The samples were then immersed in a 4-MBN solution for 2 h, followed by Raman measurements. The experimental procedure and results are shown in fig. S5H and S5I, respectively. Due to the short immersion time, 4-MBN did not fully replace the BPT molecules inside the cavity, and both BPT and 4-MBN Raman peaks were observed. Although BPT and 4-MBN are both thiol-containing molecules with nearly identical binding affinities to gold, 4-MBN was still able to partially replace BPT molecules within the gap. This result suggests that the molecular exchange method is not solely governed by binding energy, but is also influenced by factors such as molecular competition and diffusiondynamics (*41, 81*).

2D colormap of electric field distributions of LAP and TCP modes

To more clearly illustrate the mode profile used in Fig. 2 of the main text and its relation to the material boundaries, we provide a 2D representation in fig. S6, where the PVP boundaries are indicated by dashed lines.

Time-dependent SERS of single 70 nm NCoM nanogap

To verify the universality and reproducibility of the molecular kinetics within the nanogaps, we monitored the time-dependent SERS intensity evolution on multiple independent 70 nm NCoM structures. As representative examples, Fig. S7 A and B display the temporal evolution of the normalized Raman peak intensity of BPT (ring breathing mode at $\sim$ 1280 cm^{-1}) for two distinct nanoparticles during the immersion process. It is noteworthy that despite the inherent variations in absolute signal intensities among individual nanoparticles, the normalized kinetic trends remain highly consistent across different particles and excitation wavelengths.

Gap mode analysis via polarization-dependent SERS and DF spectroscopy

The DF scattering and Raman spectra of the 70 nm NCoM structure were measured under oblique incidence for both s- and p-polarized light. The Raman spectra are shown in figs. S8A and B for both 660 nm and 785 nm excitation. Raman signals were observed under p-polarization, whereas the Raman intensity under 660 nm s-polarized excitation was nearly zero. This indicates that p-polarized incidence can couple more effectively with the nanocavity plasmonic mode. The oblique incidence DF scattering spectra are shown in fig. S8C. There are notable differences between the scattering spectra of s-polarization and p-polarization, and the scattering characteristics of p-polarization coincide with the position of Raman enhancement, further proving that Raman enhancement is closely related to the polarization-dependent plasmonic coupling.

Engineering open sensing sites via partial etching with $NaBH_4$

To investigate the role of molecular channels in the molecule exchange method, 150 nm Au NPoM samples were fabricated with BPT molecules serving as the channel species, and the experimental procedure is illustrated in fig. S9A. Subsequently, the samples were treated with a 0.5 mg/mL $NaBH_4$ solution for 5 s, followed by ethanol rinsing and drying under a nitrogen stream. Since the binding affinity between hydride and gold is stronger than that between thiol and gold, $NaBH_4$ preferentially removes BPT molecules from the Au surface (*41, 82*). Given the short treatment time, some BPT molecules may have remained within the nanogap.

The samples were then immersed into 4-MBN solution 2 h, 4-MBN molecules were allowed to enter the nanogap without fully displacing the remaining BPT molecules. Raman spectroscopy was conducted at each stage of the process, and the results are presented in fig. S9C. Comparison between spectra 1 and 2 reveals a substantial decrease in the intensity of BPT characteristic peaks, indicating that $NaBH_4$ treatment successfully removed a portion of the BPT molecules from the gap. Comparison between spectra 2 and 3 revealed that the BPT peaks remained largely unchanged, while the 4-MBN characteristic peaks became clearly visible. This demonstrates that 4-MBN molecules had entered the nanogap but had not completely replaced the BPT molecules due to the controlled immersion duration.

Similarly, the samples were immersed in a 0.5 mg/mL $NaBH_4$ solution for 1 hour, during which $NaBH_4$ completely removed the BPT molecules from the cavity, effectively closing the molecular channel. The samples were then immersed in a 4-MBN solution for 24 h. As shown in fig. S9D, the Raman signal remained nearly absent, indicating that once the molecular channel is closed, the probe molecules are unable to enter the nanogap even after prolonged immersion in the solution.

To quantitatively validate the efficacy of the etching strategy, we performed a comparative study between etched and untreated NPoM structures (with BPT spacer). The SERS performance was evaluated via the molecular exchange of 4-MBN (10^{-7} M) under two distinct conditions: Specifically, both groups were immersed in 4-MBN for 5 hours, with the etched samples processed at room temperature (25 °C) and the untreated samples incubated at an elevated temperature (50 °C). As showed in fig. S10, the $NaBH_4$-etched samples processed at room temperature exhibited a SERS response more than 20-fold stronger than that of the untreated counterparts, despite the thermal

assistance applied to the latter. These results substantiate that the chemical etching treatment effectively creates highly active sites, thereby dramatically enhancing molecular exchange efficiency and sensing sensitivity.

Statistical framework for digital SERS analysis at low concentrations

Digital surface-enhanced Raman scattering has recently emerged as a transformative strategy for chemical analysis at ultralow concentrations. Unlike conventional analog SERS, where signal intensities suffer from stochastic single-molecule fluctuations and poor reproducibility at low analyte levels, digital approaches convert spectral responses into binary events ("1" or "0"), thereby enabling robust single-molecule counting (*64, 83*).

A key prerequisite for such digital conversion is a rigorous method to decide whether a measured signal should be counted as "1" or "0", so it is necessary to establish a reliable criterion to avoid misjudgments caused by background noise fluctuations. In particular, when applying the positive voxel-based determination method, the choice of threshold is critical. If the threshold is set too low, false positives are likely to occur; if it is set too high, genuine signals may be missed. Therefore, an appropriate threshold must be defined to control the false positive rate within an acceptable range.

Under the present experimental conditions, the number of test samples for each concentration exceeds 1000. To ensure the overall reliability of the data, the false positive probability for a single voxel should be less than 1/1000, namely:

$$P_{\text{false positive}}^{\text{per voxel}} < \frac{1}{1000} = 0.001$$

Assuming that the background noise follows a normal distribution:

$$I \sim \mathcal{N}(\mu_{\text{noise}}, \sigma_{\text{noise}}^2)$$

On this basis, we define the threshold I_{th} as:

$$I_{\text{th}} = \mu_{\text{noise}} + n\sigma_{\text{noise}}$$

Here, n represents a multiple of the standard deviation, and it is required that $P(I > I_{\text{th}}) < 0.001$. According to the standard normal distribution table, when $P < 0.001$, the corresponding $n > 3.1$. Therefore, in this study we set $n = 4$, i.e., the threshold is taken as the mean background signal plus four times the standard deviation. In this way, the false positive rate for each voxel is controlled to be less than 0.05%, which satisfies the reliability requirements of the experiment (*64*).

To evaluate the capability of this structure for low-concentration molecular detection, the pretreated samples were immersed in 4-MBN solutions with concentrations ranging from 10^{-8} M to

10^{-11} M for 12 hours. After immersion, the samples were rinsed with ethanol and dried with nitrogen flow. Subsequently, particle recognition and automatic measurement were performed, and for each concentration Raman signals of 225 NPoM structures were continuously obtained, and the 4-MBN characteristic peak at 2227 cm^{-1} was extracted. For this characteristic peak signal, we adopted a digitization method: when the Raman intensity was higher than the threshold, it was recorded as "1", and when it was lower than the threshold, it was recorded as "0". The digitization results are shown in fig. S12. Furthermore, the Raman test results at different concentrations were constructed into digitized Raman mapping, as shown in fig. S13. The Raman spectra at concentrations of 10^{-6} M and 10^{-8} M are shown in fig. S11.

Evaluation of relative Raman cross-sections of BPT and 2-NT

To experimentally determine the relative Raman scattering cross-sections (or Raman activities) of BPT and 2-NT within the NPoM gap, we conducted a competitive adsorption experiment. CTAC-capped Au NPoM samples (150-nm-diameter) were incubated in a mixed solution containing both BPT and 2-NT for 12 hours. The mixture was prepared at a 1:1 molar ratio, with the concentration of each component maintained at 1 mM. This equimolar condition allows us to assume comparable surface coverage for both thiol-based molecules within the nanogap. Following incubation, the samples were thoroughly rinsed with ethanol and dried under a stream of nitrogen gas (N_2) to remove any physisorbed molecules. SERS spectra were collected from 20 randomly selected NPoM structures to ensure statistical representativeness. As shown in fig. S14, the resulting average spectrum is plotted with the standard deviation indicated by the shaded error bands. Quantitative analysis was performed via Lorentzian fitting of the characteristic peaks at 1280 cm^{-1} (BPT) and 1380 cm^{-1} (2-NT). The analysis yielded an integrated peak area ratio of approximately 1.5:1 ($I_{BPT} : I_{2NT}$). Since both molecules are subjected to an identical local electromagnetic field environment within the same nanogap, the observed intensity ratio directly reflects the ratio of their intrinsic Raman cross-sections. Consequently, we determine that the relative Raman cross-section ratio η is ~1.5 in Equation 1 and 3 in the main text.

Theoretical model for spatially resolved molecular dynamics

This section provides a detailed explanation of the kinetic model used to simulate the spatiotemporally resolved molecular replacement process, as presented in Fig. 3 of the main text. The model allows for extracting the molecular spatial distributions of original and new molecules within the nanogap from the WM-SERS intensity ratios. The theoretical SERS intensity, I_s^{λ}, for a molecular species s (where $s \in \{\text{new}, \text{orig.}\}$) under an excitation wavelength λ is calculated based on the classical electromagnetic "E^4" approximation, as formulated in Equation 2 of the main text:

$$I_s^{\lambda}(r_{\text{orig.}}, \Delta r) \propto \left[\iint_A |\mathbf{E}_{\lambda}(\mathbf{r})| \cdot \omega_s(r, \Delta r)\, \mathrm{d}^2 r\right]^2 \cdot \left[\iint_A |\mathbf{E}_{\lambda_{\text{em}}}(\mathbf{r})| \cdot \omega_s(r, \Delta r)\, \mathrm{d}^2 r\right]^2 \quad \text{(S1)}$$

where the integration is performed over the entire field distribution area $A = \pi r^2$ where r = 100 nm. The term $\mathbf{E}_{\lambda,\lambda_{\text{em}}}(\mathbf{r})$ represent the spatial distributions of the near-field enhancement at the excitation wavelength (λ) and the corresponding Raman emission wavelength (λ_{em}), respectively. For the analysis in Fig. 3, the characteristic Raman peak at 1280 cm^{-1} was used. For λ = 660 nm excitation, the emission is at λ_{em} = 723 nm; for λ = 785 nm excitation, the emission is at λ_{em} = 875 nm. The corresponding simulated near-field distributions used in this calculation are shown in fig. S15.

The function $\omega_{\text{s}}(r, r_{\text{orig.}}, \Delta r)$ (or $\omega_{\text{s}}(r, r_0, \Delta r)$) is the position-dependent weighting coefficient that describes the fractional occupancy of original and new molecules $s \in \{\text{orig.}, \text{new}\}$ at a radial distance r from the nanogap center, where $\omega_{\text{orig.}}(r, r_0, \Delta r) = 1 - \omega_{\text{new}}(r, r_0, \Delta r)$. $r_{\text{orig.}}$ denotes the distance from the cavity center to the boundary of the fully replaced region; r_0 represents the distance from the cavity center to the boundary of the unreplaced region; Δr corresponds to the width of the annular transition region, where $\Delta r = r_{\text{orig.}} - r_0$. Depending on the value of Δr, $\omega_{\text{new}}(r, r_0, \Delta r)$ should be divided in to four conditions:

(i): For $\Delta r = 0$, the unchanged and replaced zones exhibit a sharp boundary without mixed zone (fig. S16C). For $r > r_{\text{orig.}}$, original molecule (BPT) is fully replaced by the new analyte (2-NT); for $r < r_{\text{orig.}}$, the original molecules remain unaffected. The weight coefficient of the new molecules as a function of r was shown as:

$$\omega_{\text{new}}(r, r_0, \Delta r) = \begin{cases} 0, & r < r_{\text{orig.}}, \\ 1, & r \geq r_{\text{orig.}}. \end{cases} \qquad (\Delta r = 0) \quad \text{(S2)}$$

(ii): For $\Delta r > 0$, $r_0 \geq 0$, the cavity can be divided into three regions (Fig. 3C in the main

text, fig. S16D): (i) unchanged region ($r < r_0$, the original remains unreplaced), (ii) mixed region ($r_0 < r < r_{orig.}$, coexistence of the original and the new, with width Δr), and (iii) replaced region ($r > r_{orig.}$, the original is fully replaced by the new). The weight coefficient of the new as a function of r in this case was shown as:

$$\omega_{new}(r, r_0, \Delta r) = \begin{cases} 0, & 0 \le r \le r_0, \\ \exp\left(n\left(\frac{r-r_0}{\Delta r} - 1\right)\right), & r_0 < r \le r_{orig.}, \\ 1, & r > r_{orig.}. \end{cases} \quad (\Delta r > 0,\ r_0 \ge 0) \tag{S3}$$

here we set the parameter $n = 3$, which defines the effective width of the exponential decay to be where the function value drops to $1/e^3$. This choice ensures a more gradual transition to zero compared to n=1 (decay to $1/e$), thereby reducing abrupt changes at the boundary of the mixed zone, as highlighted by the vertical dashed line in fig. S16D.

(iii): In the case of $\Delta r > 0,\ r_0 < 0$, the new molecules have penetrated into the nanogap center with the (i) unchanged zone disappeared, where the mixed zone with a exponential function start to overlap with its mirror result (fig. S16E). To ensure a smooth transition, a mirrored exponential function and a correction factor C are introduced, the weight coefficient is then changed as:

$$\omega_{new}(r, r_0, \Delta r) = \begin{cases} C\left[\exp\left(n\left(\frac{r-r_0}{\Delta r} - 1\right)\right) + \exp\left(n\left(\frac{-r-r_0}{\Delta r} - 1\right)\right)\right], & 0 \le r \le -r_0, \\ \exp\left(n\left(\frac{r-r_0}{\Delta r} - 1\right)\right), & -r_0 < r \le r_{orig.}, \\ 1, & r > r_{orig.}. \end{cases} \quad (\Delta r > 0,\ r_0 < 0) \tag{S4}$$

$$C = \frac{\exp\left(n\left(\frac{-nr_0}{\Delta r} - 1\right)\right)}{\exp\left(n\left(\frac{-nr_0}{\Delta r} - 1\right)\right) + \exp(-n)} \tag{S5}$$

(iv): The calculated value of $\omega_{new}(r, r_0, \Delta r)$ exceeds 1 in the central region ($r = 0$) and is capped at 1, signifying that this area is now fully saturated and has become part of the "replaced" zone (iii). The concept of a "mixed" zone (ii) becomes less relevant as the entire nanogap approaches complete replacement(fig. S16F).

Based on Equation S2-S4, I_s^λ can be calculated via equation S1. The theoretical normalized

SERS intensity ratios of the new molecules are thus obtained as:

$$R^{\lambda}_{\text{new}}(r_{\text{orig.}}, \Delta r) = \frac{I^{\lambda}_{\text{new}}}{I^{\lambda}_{\text{new}} + \eta I^{\lambda}_{\text{orig.}}}, \quad \lambda \in \{660, 785\} \tag{S6}$$

where $\eta = \sigma_{\text{BPT}}/\sigma_{\text{2NT}}$ is the ratio of the Raman cross-sections of BPT to 2-NT in the nanogap, which is estimated to be 1.5 (see fig. S14). These intensity ratios contain the information from the molecular locations. The theoretical curves in Fig. 3D of the main text are generated by plotting $R^{660}_{\text{new}}(r_{\text{orig.}}, \Delta r)$ against $R^{785}_{\text{new}}(r_{\text{orig.}}, \Delta r)$. Each colored curve corresponds to a fixed transition width, Δr (ranging from 0 to 100 nm). To trace a single curve, the infiltration progress parameter, $r_{\text{orig.}}$, is swept from 0 to a value larger than the nanocube radius (e.g., 100 nm). This results generates a trajectory in the (R^{785}_{new}, R^{660}_{new}) plane, which effectively captures the trend observed in the experimental data.

To further validate the robustness of our analysis and assess the impact of theoretical approximations, we compared the rigorous SERS model (Eq. S1, utilizing both excitation and emission fields) with the widely used $|E|^4$ approximation (the "E4 model") (*14, 62*). In this simplified framework, the field distribution at the Raman emission wavelength is assumed to be identical to that at the excitation wavelength, ignoring the spectral shift of the plasmon resonance. Consequently, Eq. S1 is simplified as follows:

$$I^{\lambda}_{s}(r_{\text{orig.}}, \Delta r) \propto \left[\iint_{A} |\mathbf{E}_{\lambda}(\mathbf{r})| \cdot \omega_{s}(r, \Delta r)\, \mathrm{d}^2 r \right]^4 \tag{S7}$$

Using this approximation, we calculated the theoretical trajectories using only the near-field distribution patterns at $\lambda = 660$ nm and 785 nm. The resulting correlation plot is presented in fig. S17B. It is evident that even with the simplified E4 model, the overall trend remains consistent with the experimental results. The primary discrepancy lies in the quantitative estimation of the mixed region width (Δr), suggesting that while the E4 model captures the qualitative physics, the rigorous inclusion of emission fields (Eq. S1) is necessary for precise spatial quantification.

Furthermore, we extended the SERS model based on Eq. S1 to investigate the potential impact of non-ideal infiltration pathways. While the primary model assumes isotropic radial infiltration, local variations may induce asymmetry. To test this, we constructed an asymmetric infiltration model where molecules preferentially penetrate and replace the spacer layer from the bottom-left corner of the nanocube propagating towards the top-right corner. The simulation results are plotted

in fig. S17C. The overall trajectory aligns well with the experimental distribution. Notably, data points in the upper-right region of the plot, which showed larger deviations from the symmetric prediction (fig. S17A), exhibit improved agreement with the asymmetric model in fig. S17C. This observation supports the hypothesis that in realistic experimental scenarios, molecular infiltration likely proceeds via a superposition of pathways, where probabilistic unidirectional infiltration plays a role alongside symmetric radial diffusion.

Kinetic analysis of sequential molecular exchange in the plasmonic nanogap: a Langmuir model

To quantitatively elucidate the molecular exchange dynamics illustrated Fig. 1E and Fig. 6G in the main text , we employed a time-dependent Langmuir kinetic model. This model provides a robust framework for describing the adsorption and desorption processes governing the replacement of molecules within the plasmonic nanogaps.

Specifically, the kinetics of molecular adsorption into a surface (an nanogap here) can be described by the simplified exponential rise equation derived from the Langmuir model: (*44, 54, 84, 85*).

$$\sigma(t) = A[1 - \exp(-kt)] \tag{S8}$$

where $\sigma(t)$ represents the fractional surface coverage of the incoming molecule at time t. The parameter A is the equilibrium coverage fraction, defined as $A = k_a c/(k_a c + k_d)$, where k_a is the adsorption rate constant, k_d is the desorption rate constant, and c is the solute concentration. For the purpose of fitting the adsorption phases shown in Fig. 1E, where incoming molecules fully replace the resident ones, we assume an irreversible adsorption process ($k_d \ll k_a c$), which simplifies the equilibrium coverage to $A \approx 1$. The term k is the apparent rate constant, which dictates the speed of the exchange process and is defined as $k = (k_a c + k_d)/N$, where N is the maximum surface adsorption density at saturation. The characteristic time constant for the process is given by $\tau = 1/k$ (unit s^{-1}). Conversely, the desorption process, which corresponds to the decay of the SERS signal from the resident molecule being replaced, can be modeled as a first-order decay:

$$\sigma_{\text{des}}(t) = \sigma_{\text{eq}} \exp(-k't) \tag{S9}$$

where $\sigma_{\text{des}}(t)$ is the surface coverage of the desorbing species, σ_{eq} is its initial equilibrium coverage, and k' is the apparent desorption rate constant.

We applied this kinetic framework to fit the normalized SERS intensity data presented in Fig. 1E in the main text. Each adsorption phase (the signal rise of MB, BSe, and BPT) was fitted using Equation S8 with A fixed at 1, while the desorption phases (the signal decay of PVP, MB, and BSe) were fitted using Equation S9. The maximum surface adsorption densities (N) for each molecule were determined from literature values and our experimental estimates: BPT, 5.8×10^{-10} mol $\cdot$ cm^{-2}; BSe,

6.9×10^{-10} mol · cm^{-2}; MB, 2.3×10^{-10} mol · cm^{-2}; and PVP, 5.0×10^{-12} mol · cm^{-2}. The extracted kinetic parameters are systematically compiled in table S2. This quantitative analysis provides a deeper understanding of the molecular exchange and binding affinities within the nanochannel.

The kinetic parameters in table S2 reveal substantial differences in the infiltration and binding behavior of the molecules. First, the desorption of the initial PVP spacer is exceptionally rapid ($k' = 3.17 \times 10^{-1}$ s^{-1}]), approximately two orders of magnitude faster than the adsorption rates of the subsequent probe molecules. This indicates that the PVP layer is highly labile and is readily displaced, confirming its role as an effective, temporary spacer that facilitates the opening of the nanochannel for subsequent analyte infiltration. For the probe molecules, the sequential replacement process dictates the observed adsorption kinetics. The adsorption rate constants (k_a) follow the order: MB (9.55×10^{-6} cm^{-1}) > BSe (2.27×10^{-6} cm^{-1}) > BPT (1.25×10^{-6} cm^{-1}). This trend is governed by the relative binding affinities between the incoming molecule and the resident molecule it displaces. The initial replacement of weakly-bound PVP by MB is highly favorable due to the large difference in their binding energies, resulting in the largest measured k_a. Subsequently, the exchange of MB with BSe becomes more challenging as their binding affinities are more comparable, leading to a smaller k_a for BSe. The final replacement of BSe by BPT is the slowest, as both are thiol-based molecules with very similar binding energies, resulting in the smallest kinetic driving force and the lowest k_a.

For the real-time microfluidic data in Fig. 6G in the main text, which tracks the replacement of CTAC by BPT, the data were fitted using Eq. S8. The best-fit parameters were obtained as $A = 0.788$, $k = 8.41 \times 10^{-3}$, $k_a = 3.84 \times 10^{-3}$ cm · s^{-1} and $k_d = 1.03 \times 10^{-12}$ mol · cm^{-2} · s^{-1}. A compelling comparison arises from the microfluidic experiment , where BPT replaces CTAC-a surfactant bound by relatively weak physisorption. The fitted adsorption rate constant for this process ($k_a = 3.84 \times 10^{-3}$ cm · s^{-1}) is three orders of magnitude larger than that for BPT replacing BSe ($k_a = 1.25 \times 10^{-6}$ cm · s^{-1}). This stark difference underscores the critical role of the resident molecule's binding strength.

Collectively, this quantitative kinetic analysis provides compelling evidence for the sequential, affinity-driven molecular exchange process within the plasmonic nanochannel. The distinct adsorption and desorption rates for each species not only validate the proposed mechanism but also highlight the platform's capability to differentiate between analytes based on their dynamics and

binding properties at the nanoscale.

To further probe the general applicability of this mechanism, we investigated the reverse scenario: replacing a strongly-bound molecule (BPT) with one known to have a weaker binding affinity (MB). For this experiment, CTAC-capped 80-nm-diameter NPoMs were first saturated with BPT, and then immersed in an MB solution under 70 °C heating. As shown in fig. S18, after just 1 hour, the characteristic MB peak at 446 cm^{-1} appeared, while the BPT signal at 1280 cm^{-1} weakened to less than half its original intensity. After 12 hours, the BPT peak almost completely disappeared, while the MB peak became approximately four times stronger, indicating a near-complete replacement. This result confirms that reverse infiltration by a weaker-binding molecule is kinetically possible, although substantially slower than the thiol-for-thiol exchange. It provides strong evidence that our nanochannel functions as a general, open system that facilitates bidirectional exchange, rather than one that relies exclusively on a strong-for-weak replacement process to create access.

High-resolution STEM validation of the sub-2-nm plasmonic nanochannel

We performed cross-sectional characterization to explicitly validate the formation of the ultrathin nanogap within the NPoF architecture used in Fig. 6 in the main text (see schematics in fig. S19A). The cross-sectional lamella was prepared using a focused ion beam (FIB) lift-out technique (Thermo Fisher, Helio Nanolab G3 UC). Initially, the region of interest was protected by depositing a 100-nm-thick carbon layer via low-voltage electron beam-induced deposition to prevent surface damage, followed by a several-micrometer-thick platinum layer via ion beam-induced deposition. The sample was then milled into a 2-μm-thick membrane, lifted out, mounted onto a transmission electron microscopy (TEM) grid, and subsequently thinned to approximately 100 nm. The resulting cross-sectional lamella was conducted using a high-angle annular dark-field (HAADF) scanning transmission electron microscopy (STEM, JEOL JEM-ARM300F GRAND ARM).

As shown in fig. S19B, the cross-sectional view clearly reveals the NPoF junction. Quantitative analysis of the vertical intensity profile across the nanogap (fig. S19C), obtained by averaging gray values horizontally within the marked region to enhance the signal-to-noise ratio, yields a gap height of approximately 1.3 nm. Specifically, to rigorously quantify the gap size (g) and differentiate the true physical gap from the projection of the spherical nanoparticle boundaries, we utilized an inflection-point method. The boundaries were identified at the positions where the signal intensity exhibits the sharpest transition (maximum curvature) from the gap floor to the walls. Based on this analysis, the gap region is defined between the coordinates of 12.8 nm and 14.1 nm in the intensity profile (see fig. S19), yielding a net thickness of $g \approx 1.3$ nm. This approach avoids the overestimation inherent in FWHM measurements caused by the projection of the particle's curvature.

It is worth noting that the slight curvature observed in the Au foil arises from the mechanical deformation of the underlying optical adhesive (NOA61) layer during the thinning process. Additionally, the minor shape distortion of the 150-nm-diameter Au nanoparticle, which appears strictly spherical in SEM characterization (see fig. S3D), is attributed to the stress exerted by the protective carbon layer deposition. These artifacts are specific to the ex-situ preparation and do not reflect the intrinsic geometry during in-situ optical measurements.

Microfluidic chip fabrication and experimental setup

The microfluidic chip was mounted on a motorized XYZ stage and data were collected using an inverted microscope system, as shown in fig. S20A. A front-view photograph of the chip is shown in fig. S20B, and the cross-sectional schematic along with the structural dimensions is presented in fig. S20C. During the experiments, molecular solutions were introduced into the microchannels using a syringe pump to precisely regulate the fluid dynamics. A constant flow rate of 0.02 mL/s was maintained for all measurements. A photograph of one of the syringe pumps used in the experiments is shown in fig. S20D.

Stability assessment of NPoM nanostructures

The mechanical stability of the NPoM structures is critical for reliable molecular exchange dynamics. In our fabrication process, the nanoparticles are immobilized on the Au film surface through a combination of van der Waals interactions and ligand-mediated adhesion. Once deposited and dried, the vast majority of the particles remain stably attached, resisting displacement during immersion in target molecular solutions.

To quantify this stability, we performed time-dependent monitoring of 80 nm NPoM samples. As shown in fig. S21, the spatial positions of the nanoparticles remained unchanged over a 6-hour immersion period (also see Supplementary Movie 1), and their corresponding DF scattering spectra exhibited negligible variations, indicating preserved structural integrity.

Furthermore, to assess stability under fluid flow conditions, real-time monitoring was conducted within the microfluidic setup. A MB solution was injected into the channel to visualize the flow. As recorded in Supplementary Movie 2, the nanoparticles maintained their positions throughout the fluid injection process, confirming that no sliding or rolling occurred. These results demonstrate that the NPoM structures are immobilized and suitable for dynamic molecular exchange experiments.

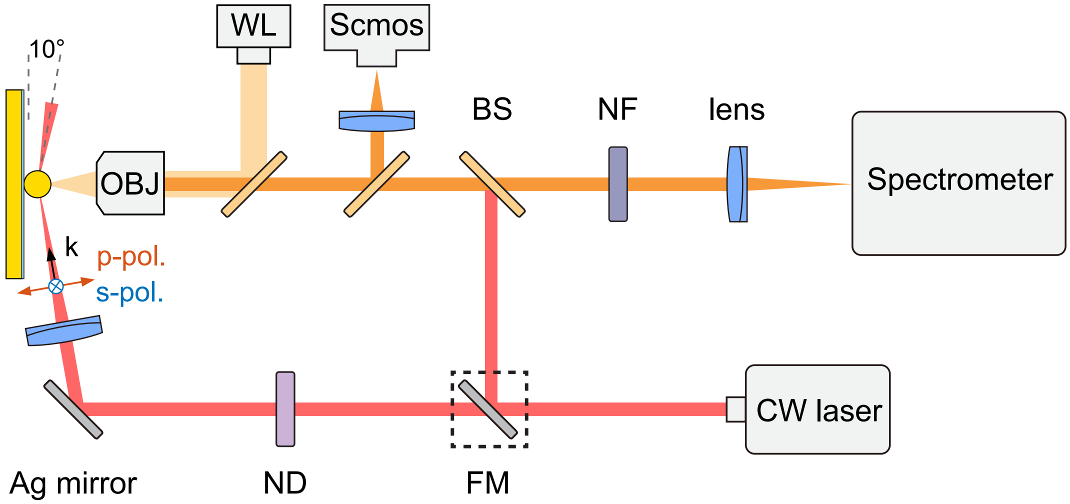

Figure S1: **Schematic of the light path for DF scattering and Raman measurement.** ND: neutral density filter; FM: flip mirror; WL: white light source; BS: beam splitter with reflection: transmission ratio (R:T) = 10:90; NF: notch filter; OBJ: Olympus objective(0.9 NA, 100 ×); Spectrometer: Princeton Instruments, grating: 300 lines/mm for Raman and 150 lines/mm for DF scattering.

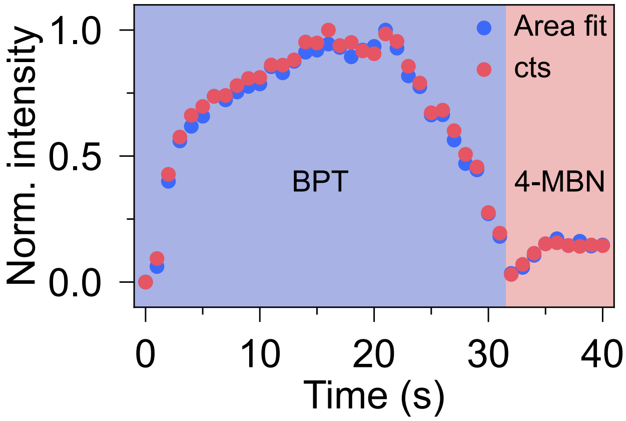

Figure S2: **Normalized Raman kinetics monitored during the microfluidic experiment.** The red dots represent the normalized peak intensity, while the blue dots represent the normalized peak area obtained via Lorentzian fitting. The monitoring tracks the BPT peak at $\sim$ 1280 cm^{-1} during the first 31 s (blue shaded area) and switches to the 4-MBN peak at $\sim$ 2227 cm^{-1} from 32 s to 40 s (red shaded area).

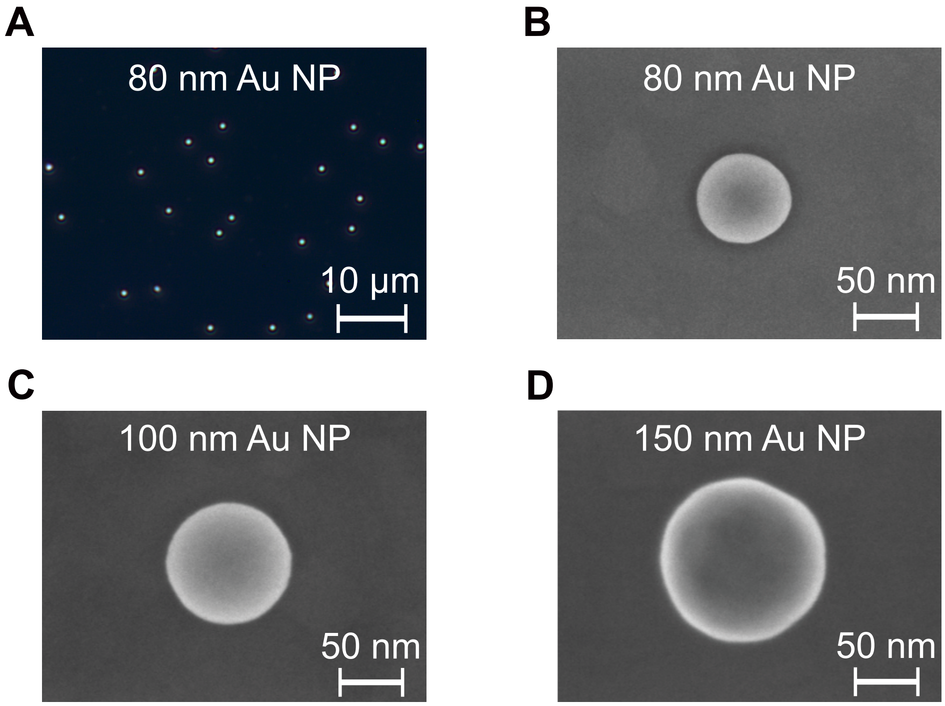

Figure S3: **DF and SEM images of the sample.** (**A**) DF images of 80 nm NPoM. (**B**)-(**D**) corresponds to 80-nm-diameter CTAC-capped Au nanospheres (CTAC), 100-nm-diameter CTAC-capped Au nanospheres and 150-nm-diameter CTAC-capped Au nanospheres, respectively.

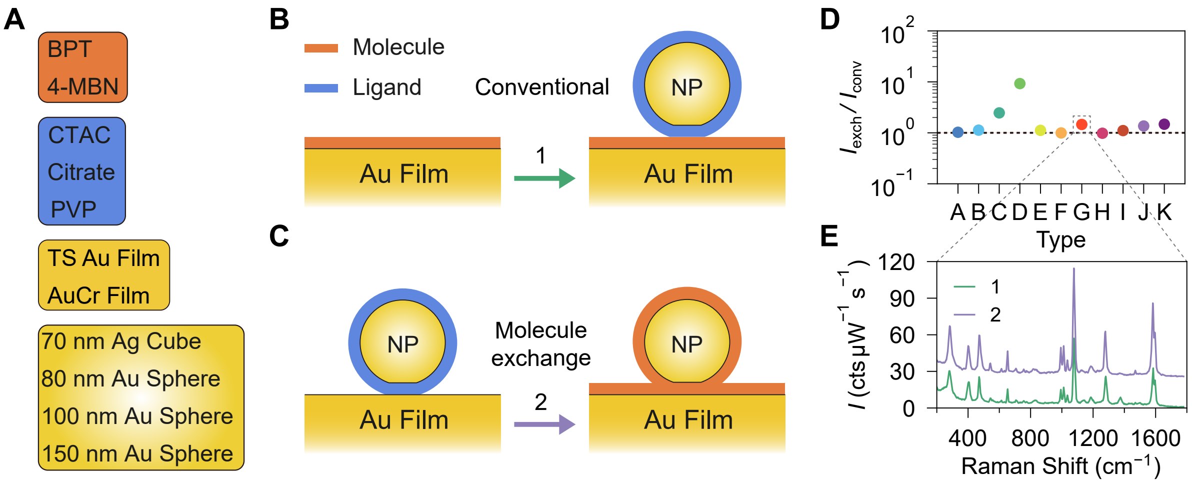

Figure S4: **Comparative SERS performance of molecular exchange versus conventional methods.** (**A**) Matrix of materials (ligands, nanoparticles, and substrates) used to construct the 11 distinct sample configurations (A–K) detailed in Table S1. (**B**, **C**) Schematic workflows for the two fabrication strategies: (B) the conventional method, where nanoparticles are deposited onto a pre-formed SAM on the Au film; and (C) the molecular exchange method, where the spacer is formed by exchanging ligands after nanoparticle deposition. (**D**) Logarithmic plot of the SERS intensity ratio of the molecular exchange method to conventional method ($I_{\text{exch}}/I_{\text{conv}}$) across all sample types. The dashed reference line (ratio = 1) demarcates the performance threshold; data points above this line indicate that the molecular exchange method yields superior enhancement. Intensities were analyzed using the 1280 cm^{-1} mode for BPT and the 1073 cm^{-1} mode for 4-MBN. (**E**) Representative Raman spectra for sample type G, displaying the signal enhancement achieved by the molecular exchange method compared to the conventional approach (averaged over nine independent particles).

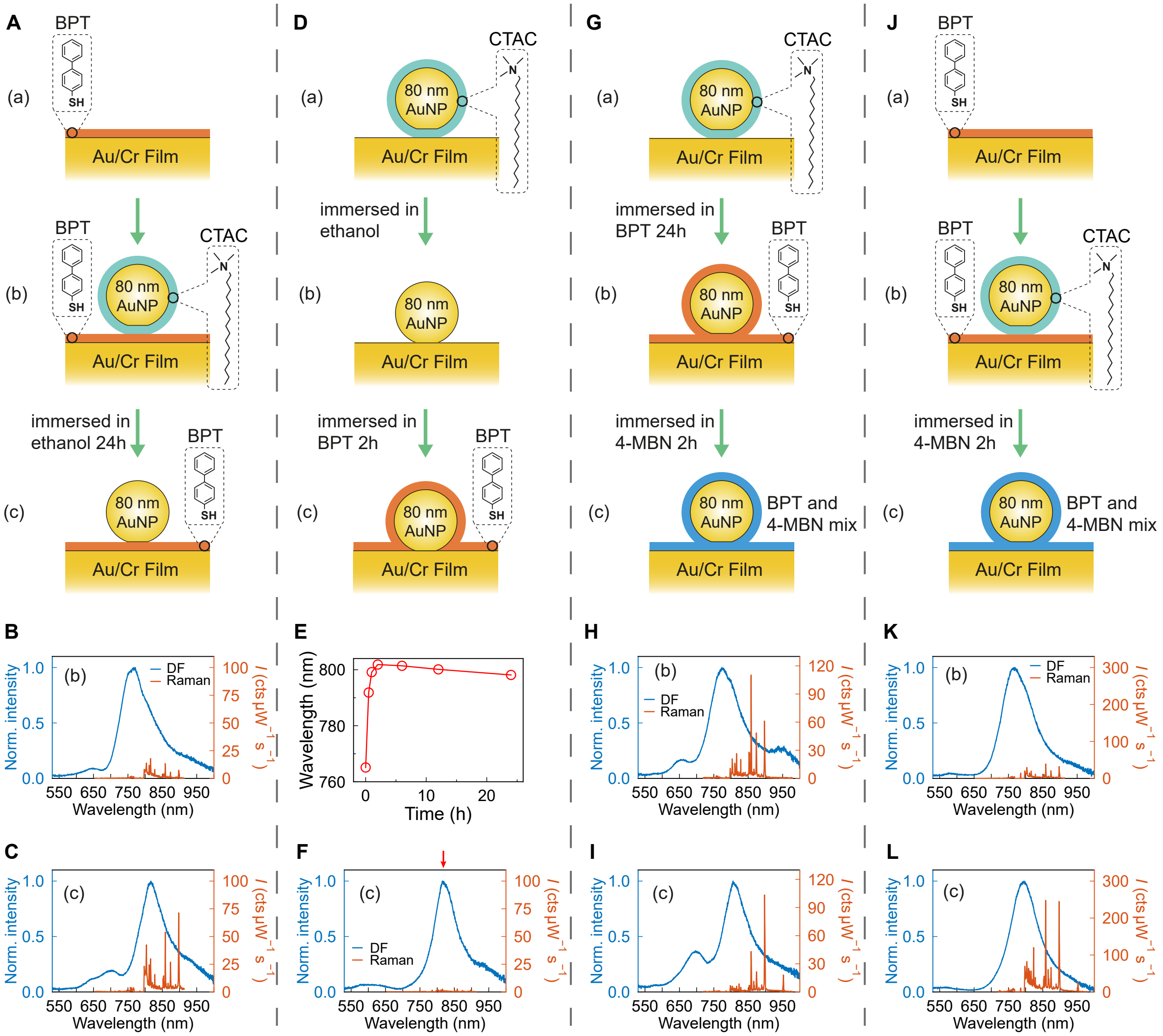

Figure S5: **Regulation of the plasmonic nanochannels.** (**A**) Schematic of nanocavity structures fabricated via the conventional method, followed by immersion in ethanol for 24 h. (**B**, **C**) Corresponding DF scattering (blue curves) and Raman spectra (brown curves) for the stages labeled (b) and (c) in (A), respectively. (**D**) Schematic of Au nanoparticles deposited on a Au film and subsequently immersed in ethanol for 0.5, 1, 2, 6, 12, and 24 h. (**E**) Time-dependent shift of the main DF scattering peak positions extracted from the immersion process in (D). (**F**) DF scattering and Raman spectra obtained after subsequent immersion in BPT solution for 2 h (red arrow indicates the peak position). (**G**) Schematic of samples prepared via the molecular exchange method followed by immersion in a 4-MBN solution for 2 h. (**H**, **I**) Comparison of spectra before (step b) and after (step c) the 4-MBN immersion described in (G). (**J**) Schematic of samples prepared via the conventional method followed by immersion in a 4-MBN solution for 2 h. (**K**, **L**) Comparison of spectra before (step b) and after (step c) the 4-MBN immersion described in (J).

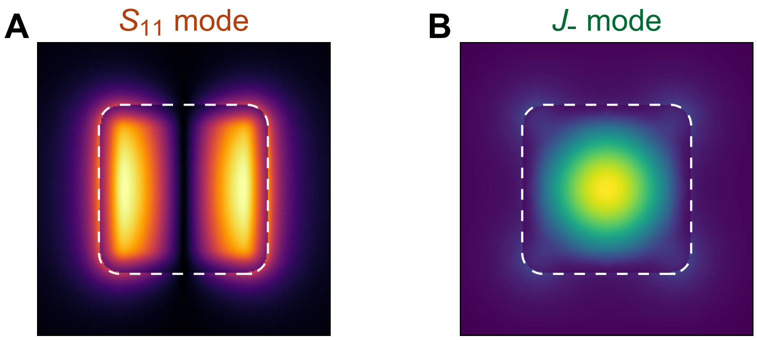

Figure S6: **Plasmonic mode patterns with PVP-capped nanocube boundary** (**A**) TCP S_{11} mode. (**B**) LAP J_{-} mode.

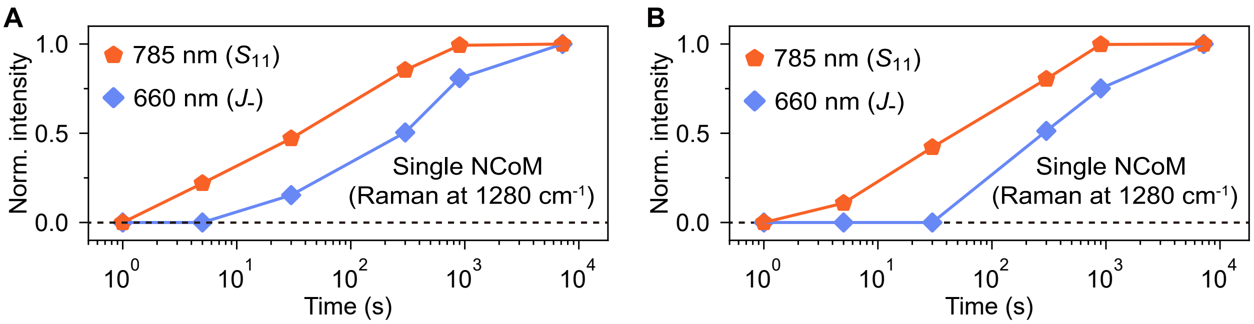

Figure S7: **Time-dependent SERS intensity evolution of individual 70 nm NCoM nanogaps.** (**A**) and (**B**) Normalized BPT Raman peak intensity at $\sim$ 1280 cm^{-1} as a function of immersion time for two independent nanoparticles.

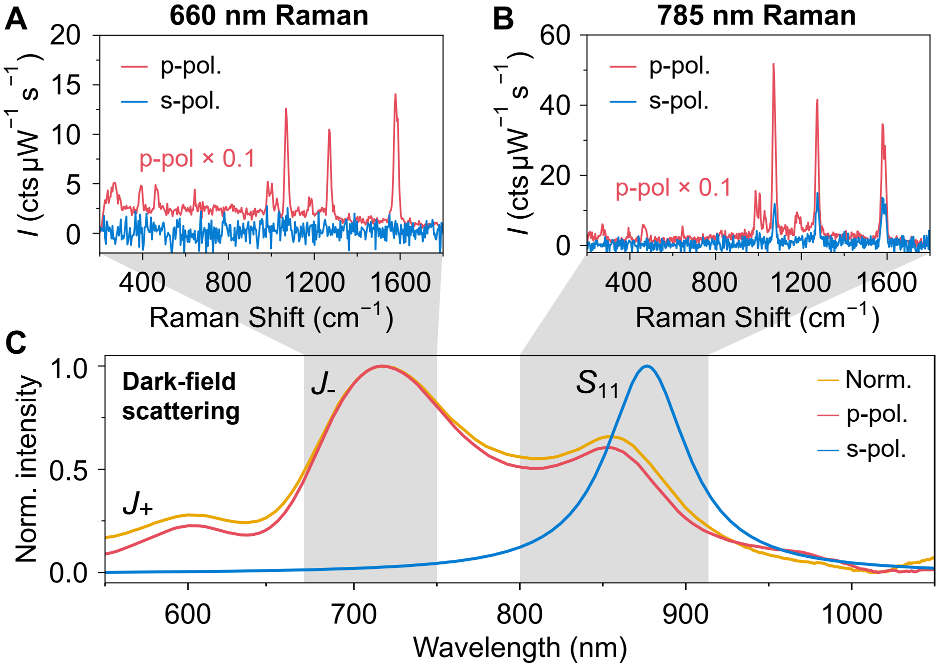

Figure S8: **Raman and DF scattering experiment of 70 nm Ag NCoM under the oblique incidence.** (**A**) The Raman spectra of oblique incidence excitation with 660 nm laser, while (**B**) Raman spectra of 785 nm laser excitation. (**C**) The DF scattering spectra of oblique incidence excitation and conventional conditions.

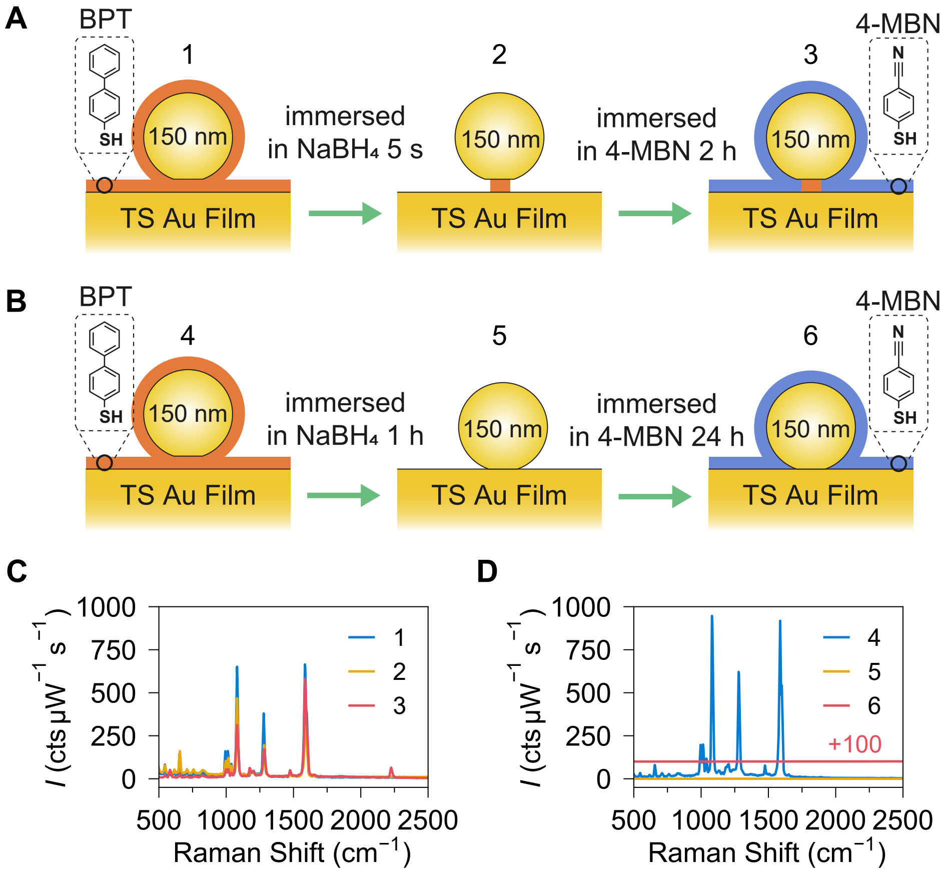

Figure S9: **Regulation of Channel Molecules by $NaBH_4$.** (**A**) Samples prepared using the molecule exchange method were first immersed in $NaBH_4$ solution for 5 s, during which the molecular channels were partially removed while BPT molecules remained in the cavity center as structural supports (step 2). The samples were then immersed in 4-MBN solution for 2 h, leading to 4-MBN molecules occupying the outer region of the cavity while BPT molecules stayed at the center (step 3). Raman spectra were performed after each step. (**B**) Samples prepared using the molecule exchange method were immersed in $NaBH_4$ solution for 1 h, resulting in complete removal of the cavity molecules (step 5), and subsequently immersed in 4-MBN solution for 24 h (step 6). (**C**) Raman spectra corresponding to the step (1-3) in process (**A**). (**D**) Raman spectra corresponding to the steps (4-6) in process (**B**).

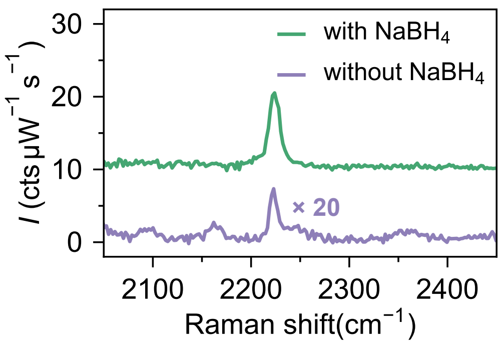

Figure S10: **Impact of $NaBH_4$ etching on 4-MBN detection.** SERS spectra showing the 4-MBN characteristic peak for the sample treated with $NaBH_4$ (green) versus the untreated sample (purple). The signal from the untreated sample is magnified 20-fold to be visible.

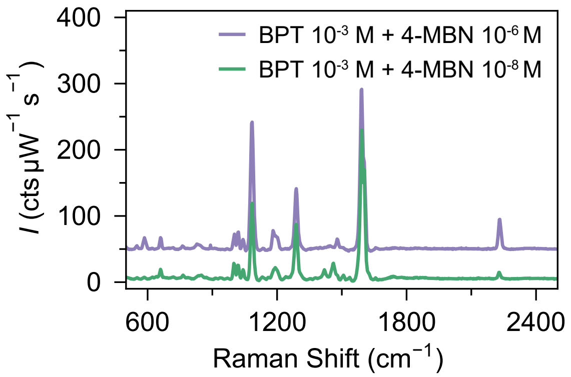

Figure S11: **Low-concentration Raman spectra of 4-MBN at** 10^{-6} **M and** 10^{-8} **M**.

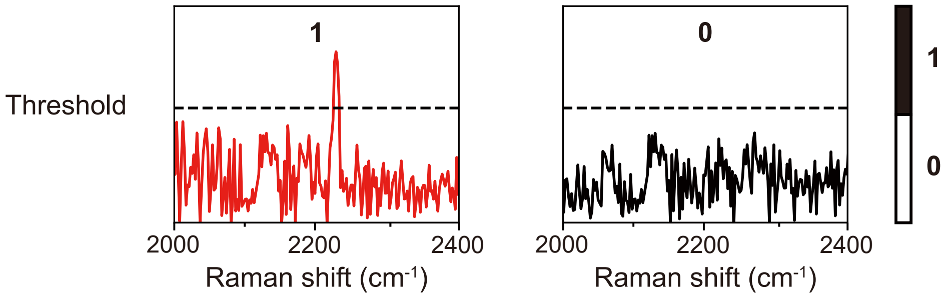

Figure S12: **Binary Criterion (0–1) for Digital Raman Mapping.** Signals above the threshold were defined as event 1, whereas signals below the threshold were defined as event 0.

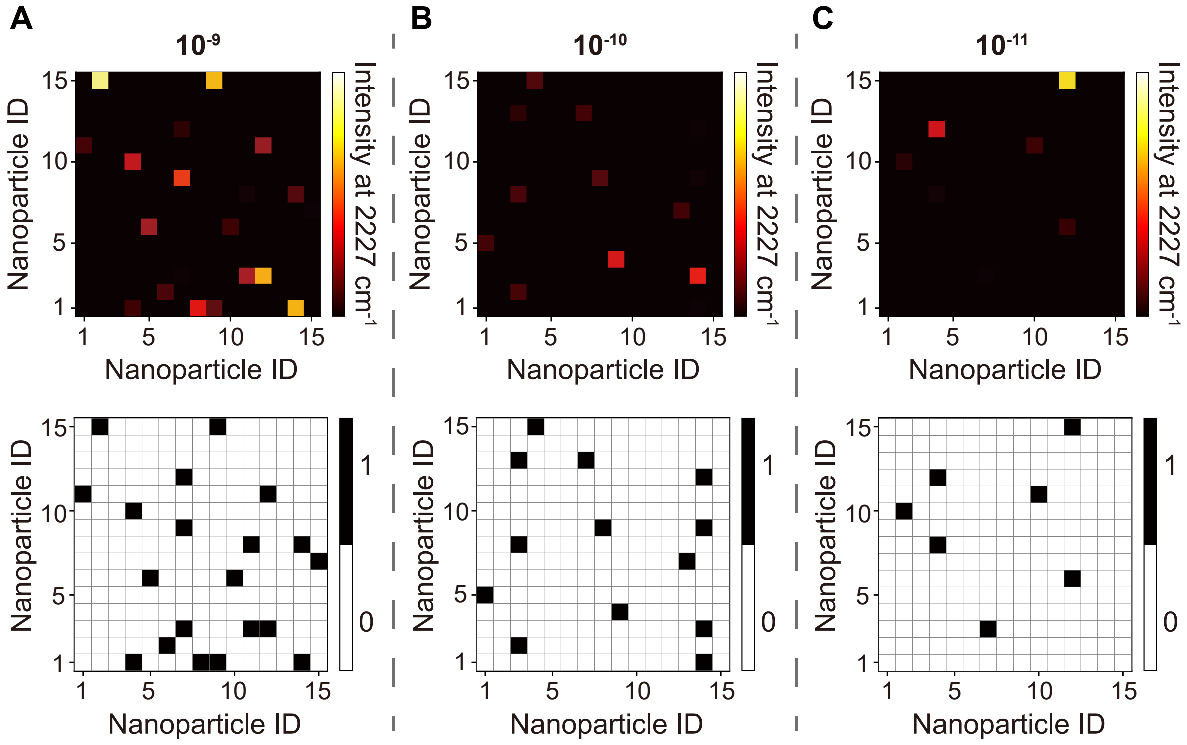

Figure S13: **Digitalized nanocavity Raman mapping.** (**A**)-(**C**) The digitized Raman map results at concentrations of 10^{-9} M, 10^{-10} M, 10^{-11} M, respectively.

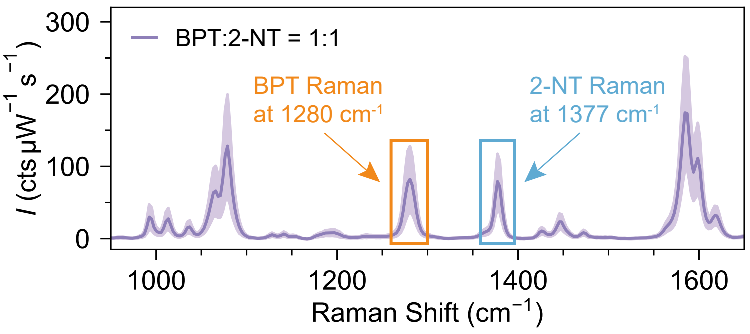

Figure S14: **SERS spectrum of BPT and 2-NT mixtures within the NPoM nanogap.** Average SERS spectrum of 150 nm NPoM samples after 12-hour incubation in a 1:1 mixed solution of BPT (1 mM) and 2-NT (1 mM). The solid line represents the mean intensity averaged over 20 randomly selected structures, while the shaded region indicates the standard deviation.

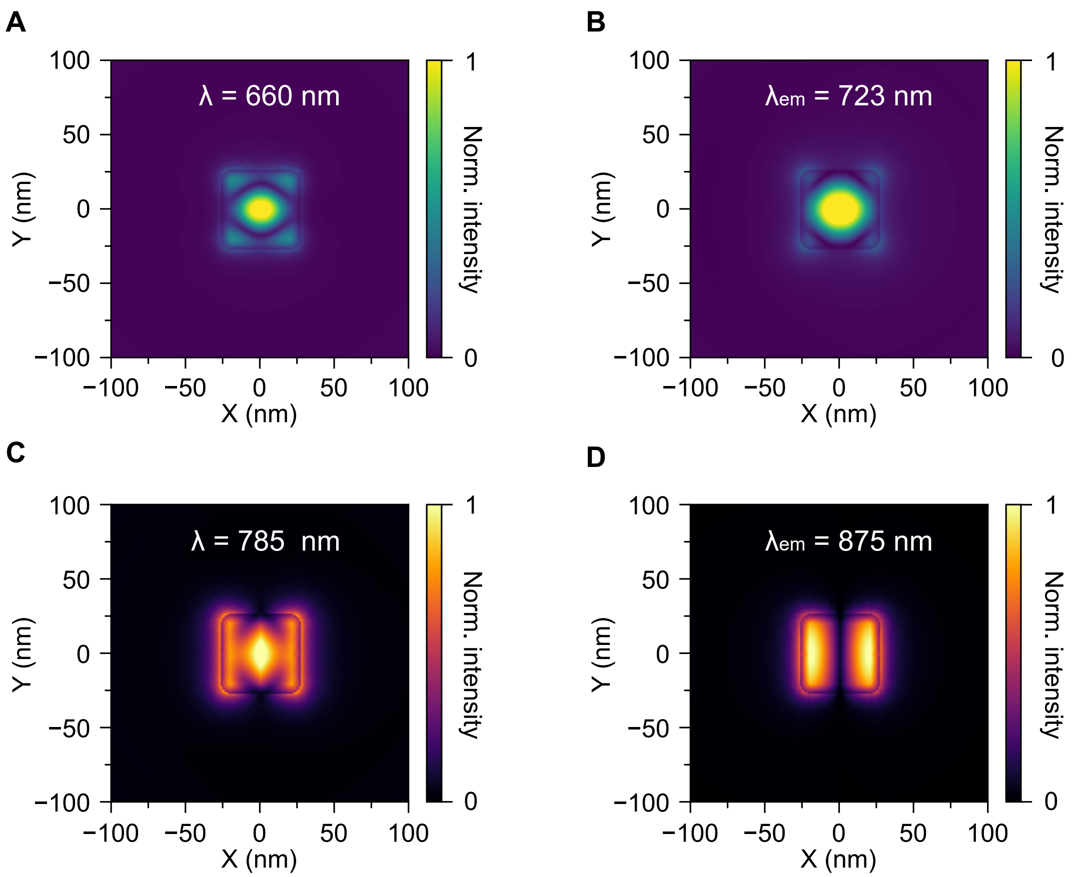

Figure S15: **The near-field distribution of the NCoM at wavelengths λ and λ_{em} .** (**A**)-(**D**) The near-field distribution under excitation at λ_{em} = 723 nm for λ = 660 nm, and at λ_{em} = 875 nm for λ = 785 nm.

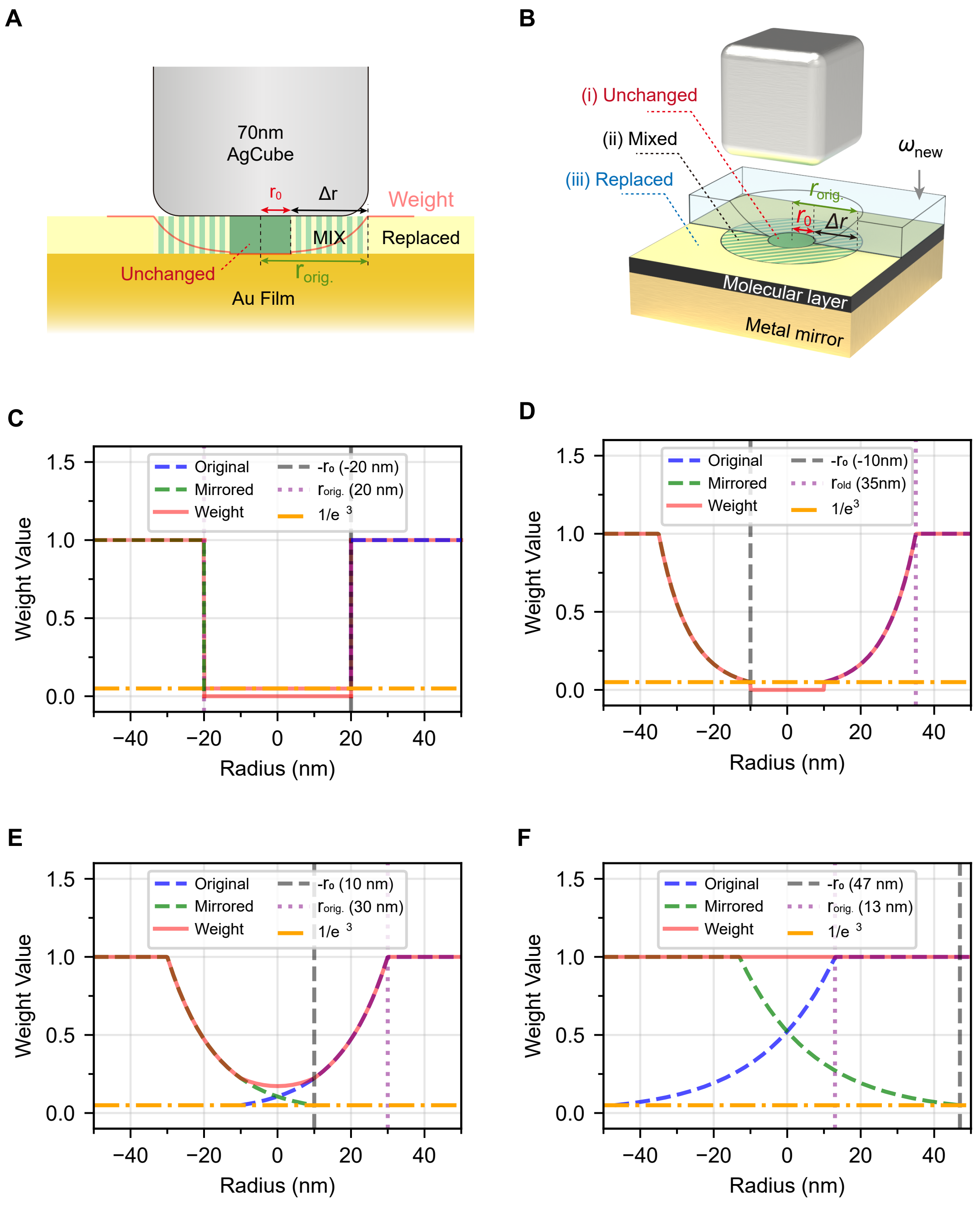

Figure S16: **Radially symmetric kinetic model.** (**A**) Schematic illustration of the radially symmetric kinetic model. (**B**) 3D schematic illustration of the model. (**C**)-(**F**) Weight Value as a function of radial position r under conditions (i) to (iv).

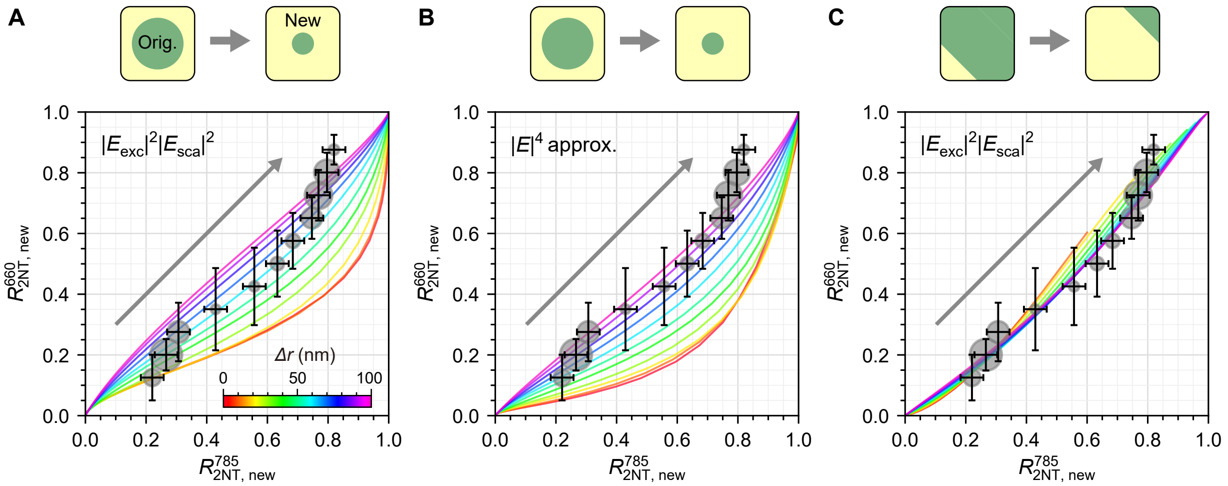

Figure S17: **Comparison of theoretical results using different infiltration and SERS models.** **(A)** Comparison between experimental data and the theoretical prediction based on the rigorous SERS model $|E_{exc}|^2|E_{sca}|^2$ (Eq. S1) combined with the symmetric radial infiltration model (reproduced from Fig. 3D in the main text). **(B)** Comparison based on the simplified E4 approximation SERS model combined with the symmetric radial infiltration model. **(C)** Comparison based on the SERS model $|E_{exc}|^2|E_{sca}|^2$ (Eq. S1) combined with an asymmetric infiltration model (unidirectional propagation from the bottom-left to the top-right corner). The asymmetric model provides a better fit for the scattered data points in the high-replacement regime (upper-right region of the plot).

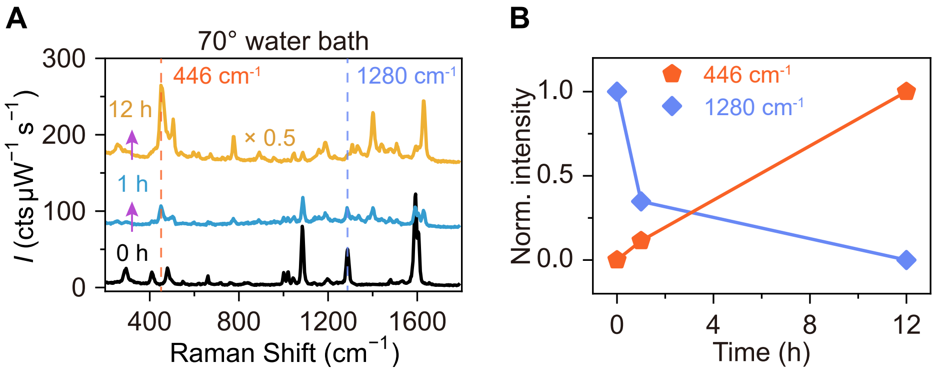

Figure S18: **Reverse molecular exchange demonstrating bidirectionality in the nanochannel.** The experiment shows the replacement of a strongly-bound BPT monolayer by a weakly-binding molecule, MB. (**A**) SERS spectra from a BPT-saturated NPoM after immersion in an MB solution for 1 h and 12 h. The gradual decrease of the characteristic BPT peak (1280 cm^{-1}) and the corresponding increase of the MB peak (446 cm^{-1}) are clearly observed. (**B**) Normalized intensity of the characteristic SERS peaks for BPT and MB as a function of immersion time.

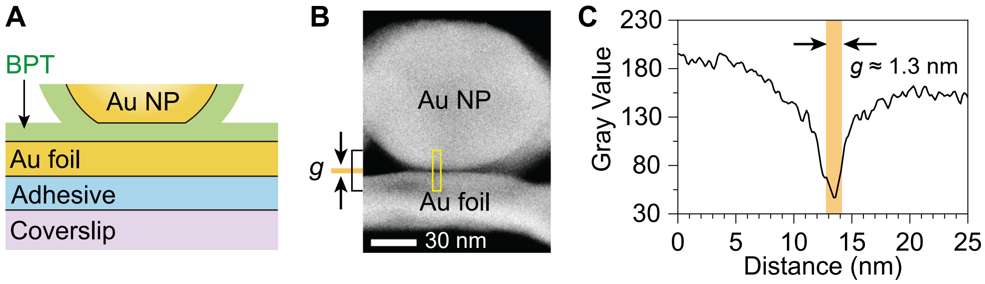

Figure S19: **High-resolution STEM validation of the sub-2-nm plasmonic nanochannel.** (**A**) Schematics of the NPoF. (**B**) Cross-sectional view of the NPoF via HAADF-STEM. (**C**) The vertical intensity profile across the nanogap from (B). The yellow shaded region indicates the gap area ($g \approx 1.3$ nm), defined between 12.8 nm and 14.1 nm. These boundaries correspond to the onset of the steep intensity rise (maximum curvature points) in the line profile, representing the physical interface while excluding the projection tail of the nanoparticle. fig. S19B is the same as Fig. 6B.

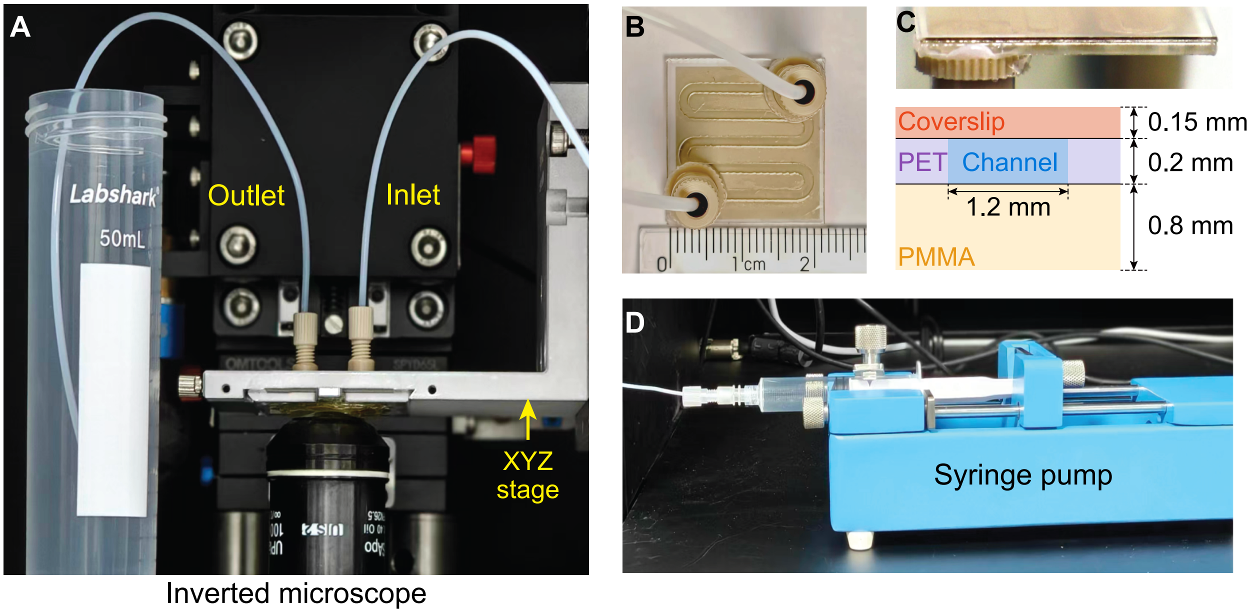

Figure S20: **Schematic illustration of the microfluidic template.** (**A**) Photograph of the testing part of the microfluidic system. (**B**) Top-view photograph of the fabricated microfluidic chip. (**C**) Cross-sectional photograph (top) and schematic (bottom) showing the channel dimensions. The structure comprises a 0.15 mm coverslip, a 0.8 mm PMMA layer, and a channel defined by a polyethylene terephthalate (PET) layer (1.2 mm wide, 0.2 mm high). (**D**) Photograph of the syringe pump used for precise fluid injection control.

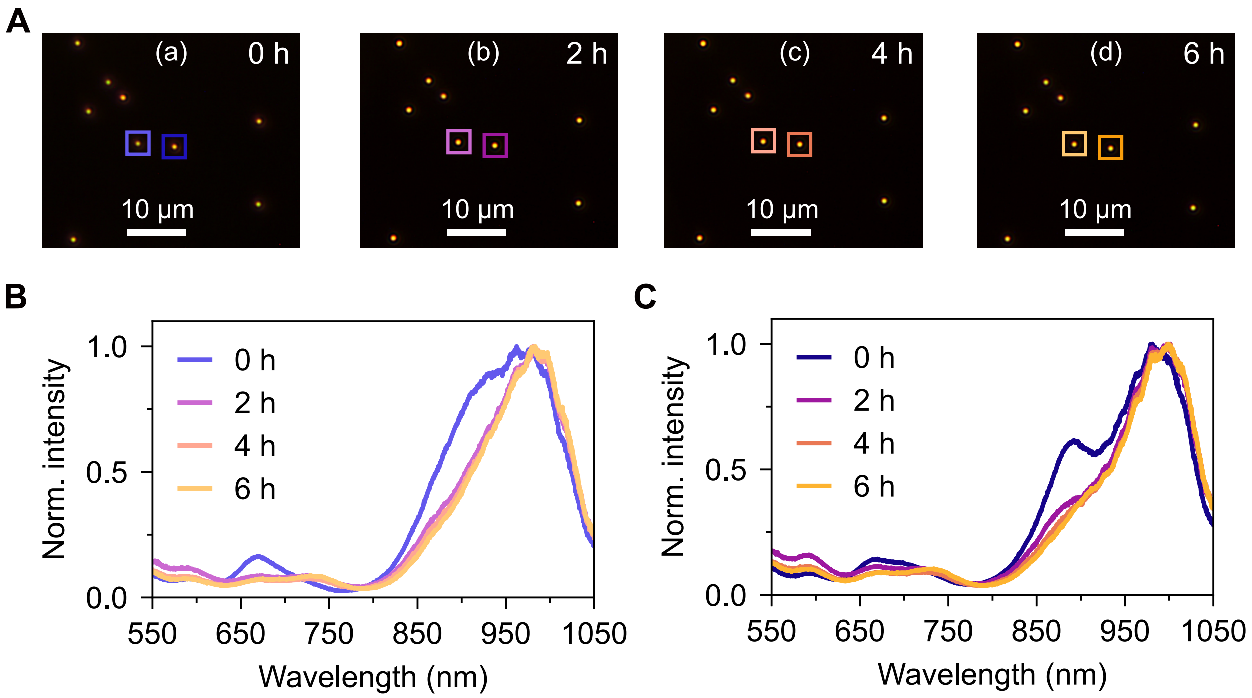

Figure S21: **Stability test of 80 nm NPoM.** (**A**) Representative DF optical images of the NPoM sample recorded at different immersion times: (a) 0 h, (b) 2 h, (c) 4 h, and (d) 6 h. Two individual nanoparticles are tracked and highlighted with colored squares. **(B)** and **(C)** Normalized DF scattering spectra corresponding to the two specific particles marked in (A) across the 6-hour immersion period. The consistent spectral features indicate high stability of the nanoparticles.

Table S1: **Composition of the different NPoM sample types.** This table summarizes the ligand, nanoparticle, channel molecule, and Au film used for each sample type.

**Type**	**Ligand**	**Nano Particle**	**Channel Molecule**	**Au Film**
A	CTAC	70 nm Ag Cube	BPT	100 nm Au/Cr film
B	CTAC	70 nm Ag Cube	BPT	TS Au film
C	PVP	70 nm Ag Cube	BPT	100 nm Au/Cr film
D	PVP	70 nm Ag Cube	BPT	TS Au film
E	Citrate	80 nm Au Sphere	4-MBN	100 nm Au/Cr film
F	Citrate	80 nm Au Sphere	BPT	TS Au film
G	CTAC	80 nm Au Sphere	BPT	100 nm Au/Cr film
H	CTAC	80 nm Au Sphere	BPT	TS Au film
I	CTAC	100 nm Au Sphere	BPT	100 nm Au/Cr film
J	CTAC	100 nm Au Sphere	BPT	TS Au film
K	CTAC	150 nm Au Sphere	BPT	100 nm Au/Cr film

Table S2: **Fitted kinetic parameters for adsorption and desorption processes.** Values of rate constants (k, k') and absorption coefficients (k_a) for different molecules.

**Molecule**	**Process**	$\boldsymbol{k}$ or $\boldsymbol{k'}$ [s^{-1}]	$\boldsymbol{k_a}$ [cm^{-1}]
PVP	Ads.	–	–
	Des.	3.17×10^{-1}	–
MB	Ads.	4.15×10^{-3}	9.55×10^{-6}
	Des.	7.41×10^{-3}	–
BSe	Ads.	3.28×10^{-3}	2.27×10^{-6}
	Des.	1.49×10^{-2}	–
BPT	Ads.	2.16×10^{-3}	1.25×10^{-6}
	Des.	–	–

Caption for Supplementary Movie S1. DF images of NPoMs before and after immersion. Shown here is an animation of DF images from the same region, demonstrating that the nanoparticles on the metal film exhibit no noticeable displacement after 2, 4, and 6 hours of immersion. Animated version of fig. S21.

Caption for Supplementary Movie S2. Real-time movie with two nanoparticles in the microfluidic channel. Here we show a movie of MB solution flowing through the nanochannel. The 150 nm nanoparticles remain in place throughout the injection process, confirming that they are not displaced or “flushed” away by the flow.

REFERENCES

1. Y. Lee, K. Choi, J.-E. Kim, S. Cha, J.-M. Nam, Integrating, validating, and expanding information space in single-molecule surface-enhanced Raman spectroscopy for biomolecules. *ACS Nano* **18**, 25359–25371 (2024).

2. J. Shen, G. Liu, Y. Han, W. Jin, Artificial channels for confined mass transport at the sub-nanometre scale. *Nat. Rev. Mater.* **6**, 294–312 (2021).

3. J. D. Spitzberg, A. Zrehen, X. F. van Kooten, A. Meller, Plasmonic-nanopore biosensors for superior single-molecule detection. *Adv. Mater.* **31**, 1900422 (2019).

4. D. Garoli, H. Yamazaki, N. Maccaferri, M. Wanunu, Plasmonic nanopores for single-molecule detection and manipulation: Toward sequencing applications. *Nano Lett.* **19**, 7553–7562 (2019).

5. G. Liu, V. Chernikova, Y. Liu, K. Zhang, Y. Belmabkhout, O. Shekhah, C. Zhang, S. Yi, M. Eddaoudi, W. J. Koros, Mixed matrix formulations with MOF molecular sieving for key energy-intensive separations. *Nat. Mater.* **17**, 283–289 (2018).

6. R. H. Tunuguntla, R. Y. Henley, Y.-C. Yao, T. A. Pham, M. Wanunu, A. Noy, Enhanced water permeability and tunable ion selectivity in subnanometer carbon nanotube porins. *Science* **357**, 792–796 (2017).

7. A. Keerthi, A. K. Geim, A. Janardanan, A. P. Rooney, A. Esfandiar, S. Hu, S. A. Dar, I. V. Grigorieva, S. J. Haigh, F. C. Wang, B. Radha, Ballistic molecular transport through two-dimensional channels. *Nature* **558**, 420–424 (2018).

8. M. Zhang, K. Guan, Y. Ji, G. Liu, W. Jin, N. Xu, Controllable ion transport by surface-charged graphene oxide membrane. *Nat. Commun.* **10**, 1253 (2019).

9. M. Lozada-Hidalgo, S. Hu, O. Marshall, A. Mishchenko, A. N. Grigorenko, R. A. W. Dryfe, B. Radha, I. V. Grigorieva, A. K. Geim, Sieving hydrogen isotopes through two-dimensional crystals. *Science* **351**, 68–70 (2016).

10. K.-G. Zhou, K. S. Vasu, C. T. Cherian, M. Neek-Amal, J. C. Zhang, H. Ghorbanfekr-Kalashami, K. Huang, O. P. Marshall, V. G. Kravets, J. Abraham, Y. Su, A. N. Grigorenko, A. Pratt, A. K. Geim, F. M. Peeters, K. S. Novoselov, R. R. Nair, Electrically controlled water permeation through graphene oxide membranes. *Nature* **559**, 236–240 (2018).

11. G.-C. Li, Q. Zhang, S. A. Maier, D. Lei, Plasmonic particle-on-film nanocavities: A versatile platform for plasmon-enhanced spectroscopy and photochemistry. *Nanophotonics* **7**, 1865–1889 (2018).

12. J. J. Baumberg, J. Aizpurua, M. H. Mikkelsen, D. R. Smith, Extreme nanophotonics from ultrathin metallic gaps. *Nat. Mater.* **18**, 668–678 (2019).

13. X. Wang, S.-C. Huang, S. Hu, S. Yan, B. Ren, Fundamental understanding and applications of plasmon-enhanced Raman spectroscopy. *Nat. Rev. Phys.* **2**, 253–271 (2020).

14. Y. Li, W. Chen, X. He, J. Shi, X. Cui, J. Sun, H. Xu, Boosting light-matter interactions in plasmonic nanogaps. *Adv. Mater.* **36**, 2405186 (2024).

15. F. Benz, M. K. Schmidt, A. Dreismann, R. Chikkaraddy, Y. Zhang, A. Demetriadou, C. Carnegie, H. Ohadi, B. de Nijs, R. Esteban, J. Aizpurua, J. J. Baumberg, Single-molecule optomechanics in “picocavities”. *Science* **354**, 726–729 (2016).

16. W. Chen, P. Roelli, A. Ahmed, S. Verlekar, H. Hu, K. Banjac, M. Lingenfelder, T. J. Kippenberg, G. Tagliabue, C. Galland, Intrinsic luminescence blinking from plasmonic nanojunctions. *Nat. Commun.* **12**, 2731 (2021).

17. H. Xu, E. J. Bjerneld, M. Käll, L. Börjesson, Spectroscopy of single hemoglobin molecules by surface enhanced Raman scattering. *Phys. Rev. Lett.* **83**, 4357–4360 (1999).

18. R. Zhang, Y. Zhang, Z. C. Dong, S. Jiang, C. Zhang, L. G. Chen, L. Zhang, Y. Liao, J. Aizpurua, Y. Luo, J. L. Yang, J. G. Hou, Chemical mapping of a single molecule by plasmon-enhanced Raman scattering. *Nature* **498**, 82–86 (2013).

19. H.-K. Choi, W.-H. Park, C.-G. Park, H.-H. Shin, K. S. Lee, Z. H. Kim, Metal-catalyzed chemical reaction of single molecules directly probed by vibrational spectroscopy. *J. Am. Chem. Soc.* **138**, 4673–4684 (2016).

20. N. H. Kim, W. Hwang, K. Baek, M. R. Rohman, J. Kim, H. W. Kim, J. Mun, S. Y. Lee, G. Yun, J. Murray, J. W. Ha, J. Rho, M. Moskovits, K. Kim, Smart SERS hot spots: Single molecules can be positioned in a plasmonic nanojunction using host–guest chemistry. *J. Am. Chem. Soc.* **140**, 4705–4711 (2018).

21. J. Langer, D. Jimenez de Aberasturi, J. Aizpurua, R. A. Alvarez-Puebla, B. Auguié, J. J. Baumberg, G. C. Bazan, S. E. J. Bell, A. Boisen, A. G. Brolo, J. Choo, D. Cialla-May, V. Deckert, L. Fabris, K. Faulds, F. J. G. de Abajo, R. Goodacre, D. Graham, A. J. Haes, C. L. Haynes, C. Huck, T. Itoh, M. Käll, J. Kneipp, N. A. Kotov, H. Kuang, E. C. Le Ru, H. K. Lee, J.-F. Li, X. Y. Ling, S. A. Maier, T. Mayerhöfer, M. Moskovits, K. Murakoshi, J.-M. Nam, S. Nie, Y. Ozaki, I. Pastoriza-Santos, J. Perez-Juste, J. Popp, A. Pucci, S. Reich, B. Ren, G. C. Schatz, T. Shegai, S. Schlücker, L.-L. Tay, K. G. Thomas, Z.-Q. Tian, R. P. Van Duyne, T. Vo-Dinh, Y. Wang, K. A. Willets, C. Xu, H. Xu, Y. Xu, Y. S. Yamamoto, B. Zhao, L. M. Liz-Marzán, Present and future of surface-enhanced Raman scattering. *ACS Nano* **14**, 28–117 (2020).

22. Y.-H. Wang, S. Zheng, W.-M. Yang, R.-Y. Zhou, Q.-F. He, P. Radjenovic, J.-C. Dong, S. Li, J. Zheng, Z.-L. Yang, G. Attard, F. Pan, Z.-Q. Tian, J.-F. Li, In situ Raman spectroscopy reveals the structure and dissociation of interfacial water. *Nature* **600**, 81–85 (2021).

23. E. Oksenberg, I. Shlesinger, A. Xomalis, A. Baldi, J. J. Baumberg, A. F. Koenderink, E. C. Garnett, Energy-resolved plasmonic chemistry in individual nanoreactors. *Nat. Nanotechnol.* **16**, 1378–1385 (2021).

24. C. Zhan, Q.-X. Wang, J. Yi, L. Chen, D.-Y. Wu, Y. Wang, Z.-X. Xie, M. Moskovits, Z.-Q. Tian, Plasmonic nanoreactors regulating selective oxidation by energetic electrons and nanoconfined thermal fields. *Sci. Adv.* **7**, eabf0962 (2021).

25. S. Hu, E. S. A. Goerlitzer, Q. Lin, B. de Nijs, V. M. Silkin, J. J. Baumberg, Alchemically-glazed plasmonic nanocavities using atomic layer metals: Controllably synergizing catalysis and plasmonics. *Nat. Commun.* **16**, 3370 (2025).

26. C. Chen, Y. Li, S. Kerman, P. Neutens, K. Willems, S. Cornelissen, L. Lagae, T. Stakenborg, P. Van Dorpe, High spatial resolution nanoslit SERS for single-molecule nucleobase sensing. *Nat. Commun.* **9**, 1733 (2018).

27. M. Belkin, S.-H. Chao, M. P. Jonsson, C. Dekker, A. Aksimentiev, Plasmonic nanopores for trapping, controlling displacement, and sequencing of DNA. *ACS Nano* **9**, 10598–10611 (2015).

28. O. N. Assad, T. Gilboa, J. Spitzberg, M. Juhasz, E. Weinhold, A. Meller, Light-enhancing plasmonic-nanopore biosensor for superior single-molecule detection. *Adv. Mater.* **29**, 1605442 (2017).

29. X. Shi, D. V. Verschueren, C. Dekker, Active delivery of single DNA molecules into a plasmonic nanopore for label-free optical sensing. *Nano Lett.* **18**, 8003–8010 (2018).

30. Y. Zhao, A. Hubarevich, A. F. de Fazio, M. Iarossi, J. A. Huang, F. de Angelis, Plasmonic bowl-shaped nanopore for Raman detection of single DNA molecules in flow-through. *Nano Lett.* **23**, 4830–4836 (2023).

31. J. Zhou, Q. Lan, W. Li, L. N. Ji, K. Wang, X. H. Xia, Single molecule protein segments sequencing by a plasmonic nanopore. *Nano Lett.* **23**, 2800–2807 (2023).

32. S. Luo, B. H. Hoff, S. A. Maier, J. C. de Mello, Scalable fabrication of metallic nanogaps at the sub-10 Nm level. *Adv. Sci.* **8**, 2102756 (2021).

33. W. Chen, S. Zhang, M. Kang, W. Liu, Z. Ou, Y. Li, Y. Zhang, Z. Guan, H. Xu, Probing the limits of plasmonic enhancement using a two-dimensional atomic crystal probe. *Light. Sci. Appl.* **7**, 56 (2018).

34. G. M. Akselrod, C. Argyropoulos, T. B. Hoang, C. Ciracì, C. Fang, J. Huang, D. R. Smith, M. H. Mikkelsen, Probing the mechanisms of large purcell enhancement in plasmonic nanoantennas. *Nat. Photonics* **8**, 835–840 (2014).

35. R. Chikkaraddy, V. A. Turek, N. Kongsuwan, F. Benz, C. Carnegie, T. van de Goor, B. de Nijs, A. Demetriadou, O. Hess, U. F. Keyser, J. J. Baumberg, Mapping nanoscale hotspots with single-molecule emitters assembled into plasmonic nanocavities using DNA origami. *Nano Lett.* **18**, 405–411 (2018).

36. H. Im, K. C. Bantz, S. H. Lee, T. W. Johnson, C. L. Haynes, S. H. Oh, Self-assembled plasmonic nanoring cavity arrays for SERS and LSPR biosensing. *Adv. Mater.* **25**, 2678–2685 (2013).

37. H.-Y. Chen, M.-H. Lin, C.-Y. Wang, Y.-M. Chang, S. Gwo, Large-scale hot spot engineering for quantitative SERS at the single-molecule scale. *J. Am. Chem. Soc.* **137**, 13698–13705 (2015).

38. A. B. Taylor, P. Zijlstra, Single-molecule plasmon sensing: Current status and future prospects. *ACS Sens.* **2**, 1103–1122 (2017).

39. W. Chen, S. Zhang, Q. Deng, H. Xu, Probing of sub-picometer vertical differential resolutions using cavity plasmons. *Nat. Commun.* **9**, 801 (2018).

40. Y. Liu, K. K. Chui, Y. Fang, S. Wen, X. Zhuo, J. Wang, Metal–organic framework-enabled trapping of volatile organic compounds into plasmonic nanogaps for surface-enhanced Raman scattering detection. *ACS Nano* **18**, 11234–11244 (2024).

41. S. M. Ansar, F. S. Ameer, W. Hu, S. Zou, C. U. Pittman Jr., D. Zhang, Removal of molecular adsorbates on gold nanoparticles using sodium borohydride in water. *Nano Lett.* **13**, 1226–1229 (2013).

42. R.-Y. Zhong, J.-W. Yang, Z. Hu, B.-Q. Xu, Removal of residual poly(vinylpyrrolidone) from gold nanoparticles immobilized on SiO_2 by ultraviolet–ozone treatment. *ACS Appl. Nano Mater.* **2**, 5720–5729 (2019).

43. C. H. Moran, M. Rycenga, Q. Zhang, Y. Xia, Replacement of poly(vinyl pyrrolidone) by thiols: A systematic study of Ag nanocube functionalization by surface-enhanced Raman scattering. *J. Phys. Chem. C* **115**, 21852–21857 (2011).

44. E. Villarreal, G. G. Li, Q. Zhang, X. Fu, H. Wang, Nanoscale surface curvature effects on ligand–nanoparticle interactions: A plasmon-enhanced spectroscopic study of thiolated ligand adsorption, desorption, and exchange on gold nanoparticles. *Nano Lett.* **17**, 4443–4452 (2017).

45. G. S. Perera, S. A. Athukorale, F. Perez, C. U. Pittman, D. Zhang, Facile displacement of citrate residues from gold nanoparticle surfaces. *J. Colloid Interface Sci.* **511**, 335–343 (2018).

46. S. Zhou, D. Huo, S. Goines, T. H. Yang, Z. Lyu, M. Zhao, K. D. Gilroy, Y. Wu, Z. D. Hood, M. Xie, Y. Xia, Enabling complete ligand exchange on the surface of gold nanocrystals through the deposition and then etching of silver. *J. Am. Chem. Soc.* **140**, 11898–11901 (2018).

47. J. Ahn, S. Shi, B. Vannatter, D. Qin, Comparative study of the adsorption of thiol and isocyanide molecules on a silver surface by in situ surface-enhanced Raman scattering. *J. Phys. Chem. C* **123**, 21571–21580 (2019).

48. A. Lapresta-Fernández, E. Nefeli Athanasopoulou, P. Jacob Silva, Z. Pelin Güven, F. Stellacci, Site-selective surface enhanced Raman scattering study of ligand exchange reactions on aggregated Ag nanocubes. *J. Colloid Interface Sci.* **616**, 110–120 (2022).

49. J.-Y. Huang, C. Zong, L.-J. Xu, Y. Cui, B. Ren, Clean and modified substrates for direct detection of living cells by surface-enhanced Raman spectroscopy. *Chem. Commun.* **47**, 5738–5740 (2011).

50. M. Viehrig, S. T. Rajendran, K. Sanger, M. S. Schmidt, T. S. Alstrøm, T. Rindzevicius, K. Zór, A. Boisen, Quantitative SERS assay on a single chip enabled by electrochemically assisted regeneration: A method for detection of melamine in milk. *Anal. Chem.* **92**, 4317–4325 (2020).

51. S. M. Sibug-Torres, D. B. Grys, G. Kang, M. Niihori, E. Wyatt, N. Spiesshofer, A. Ruane, B. de Nijs, J. J. Baumberg, In situ electrochemical regeneration of nanogap hotspots for continuously reusable ultrathin SERS sensors. *Nat. Commun.* **15**, 2022 (2024).

52. X. Wang, M. Li, L. Meng, K. Lin, J. Feng, T. Huang, Z. Yang, B. Ren, Probing the location of hot spots by surface-enhanced Raman spectroscopy: Toward uniform substrates. *ACS Nano* **8**, 528–536 (2014).

53. J. Griffiths, B. de Nijs, R. Chikkaraddy, J. J. Baumberg, Locating single-atom optical picocavities using wavelength-multiplexed Raman scattering. *ACS Photonics* **8**, 2868–2875 (2021).

54. A. Tukova, M. Tavakkoli Yaraki, A. Rodger, Y. Wang, Shape-induced variations in aromatic thiols adsorption on gold nanoparticle: A novel method for accurate evaluation of adsorbed molecules. *Langmuir* **39**, 15828–15836 (2023).

55. R. Chikkaraddy, X. Zheng, F. Benz, L. J. Brooks, B. de Nijs, C. Carnegie, M.-E. Kleemann, J. Mertens, R. W. Bowman, G. A. E. Vandenbosch, V. V. Moshchalkov, J. J. Baumberg, How ultranarrow gap symmetries control plasmonic nanocavity modes: From cubes to spheres in the nanoparticle-on-mirror. *ACS Photonics* **4**, 469–475 (2017).

56. T. Wu, D. Arrivault, W. Yan, P. Lalanne, Modal analysis of electromagnetic resonators: User guide for the MAN program. *Comput. Phys. Commun.* **284**, 108627 (2023).

57. C. Tserkezis, R. Esteban, D. O. Sigle, J. Mertens, L. O. Herrmann, J. J. Baumberg, J. Aizpurua, Hybridization of plasmonic antenna and cavity modes: Extreme optics of nanoparticle-on-mirror nanogaps. *Phys. Rev. A* **92**, 053811 (2015).

58. R. Esteban, G. Aguirregabiria, A. G. Borisov, Y. M. Wang, P. Nordlander, G. W. Bryant, J. Aizpurua, The morphology of narrow gaps modifies the plasmonic response. *ACS Photonics* **2**, 295–305 (2015).

59. A. Gopinath, E. Miyazono, A. Faraon, P. W. K. Rothemund, Engineering and mapping nanocavity emission via precision placement of DNA origami. *Nature* **535**, 401–405 (2016).

60. W. Chen, P. Roelli, H. Hu, S. Verlekar, S. P. Amirtharaj, A. I. Barreda, T. J. Kippenberg, M. Kovylina, E. Verhagen, A. Martínez, C. Galland, Continuous-wave frequency upconversion with a molecular optomechanical nanocavity. *Science* **374**, 1264–1267 (2021).

61. Z. Wang, L. Liu, D. Zhang, A. V. Krasavin, J. Zheng, C. Pan, E. He, Z. Wang, S. Zhong, Z. Li, M. Ren, X. Guo, A. V. Zayats, L. Tong, P. Wang, Effect of mirror quality on optical response of nanoparticle-on-mirror plasmonic nanocavities. *Adv. Opt. Mater.* **11**, 2201914 (2023).

62. H. Xu, J. Aizpurua, M. Käll, P. Apell, Electromagnetic contributions to single-molecule sensitivity in surface-enhanced Raman scattering. *Phys. Rev. E* **62**, 4318–4324 (2000).

63. R. Chikkaraddy, J. J. Baumberg, Accessing plasmonic hotspots using nanoparticle-on-foil constructs. *ACS Photonics* **8**, 2811–2817 (2021).

64. X. Bi, D. M. Czajkowsky, Z. Shao, J. Ye, Digital colloid-enhanced Raman spectroscopy by single-molecule counting. *Nature* **628**, 771–775 (2024).

65. H. Zhang, L. Yang, M. Zhang, H. Wei, L. Tong, H. Xu, Z. Li, A statistical route to robust SERS quantitation beyond the single-molecule level. *Nano Lett.* **24**, 11116–11123 (2024).

66. C. D. L. de Albuquerque, R. G. Sobral-Filho, R. J. Poppi, A. G. Brolo, Digital protocol for chemical analysis at ultralow concentrations by surface-enhanced Raman scattering. *Anal. Chem.* **90**, 1248–1254 (2018).

67. L. A. Warning, A. R. Miandashti, L. McCarthy, Q. Zhang, C. F. Landes, S. Link, Nanophotonic approaches for chirality sensing. *ACS Nano* **15**, 15538–15566 (2021).

68. S. A. Lee, S. Link, Chemical interface damping of surface plasmon resonances. *Acc. Chem. Res.* **54**, 1950–1960 (2021).

69. D. Ding, K. Liu, S. He, C. Gao, Y. Yin, Ligand-exchange assisted formation of Au/TiO_2 Schottky contact for visible-light photocatalysis. *Nano Lett.* **14**, 6731–6736 (2014).

70. H. Zhang, M. Jin, Y. Xia, Noble-metal nanocrystals with concave surfaces: Synthesis and applications. *Angew. Chem. Int. Ed. Engl.* **51**, 7656–7673 (2012).

71. S. Brasselet, M. D. Lew, Single-molecule orientation and localization microscopy. *Nat. Photonics* **19**, 925–937 (2025).

72. Y. U. Lee, G. B. M. Wisna, S. W. Hsu, J. Zhao, M. Lei, S. Li, A. R. Tao, Z. Liu, Imaging of nanoscale light confinement in plasmonic nanoantennas by brownian optical microscopy. *ACS Nano* **14**, 7666–7672 (2020).

73. K. A. Willets, Super-resolution surface-enhanced Raman scattering: Perspectives on the past, present, and future. *ACS Nano* **18**, 27824–27832 (2024).

74. T. A. Huijben, S. Mahajan, M. Fahim, P. Zijlstra, R. Marie, K. I. Mortensen, Point-spread function deformations unlock 3D localization microscopy on spherical nanoparticles. *ACS Nano* **18**, 29832–29845 (2024).

75. P. B. Johnson, R. W. Christy, Optical constants of the noble metals. *Phys. Rev. B* **6**, 4370–4379 (1972).

76. R. L. Olmon, B. Slovick, T. W. Johnson, D. Shelton, S. H. Oh, G. D. Boreman, M. B. Raschke, Optical dielectric function of gold. *Phys. Rev. B* **86**, 235147 (2012).

77. G. Hlawacek, I. Ahmad, M. A. Smithers, E. S. Kooij, To see or not to see: imaging surfactant coated nano particles using HIM and SEM. *Ultramicroscopy* **135**, 89–94 (2013).

78. R. T. Hill, J. J. Mock, A. Hucknall, S. D. Wolter, N. M. Jokerist, D. R. Smith, A. Chilkoti, Plasmon ruler with Ångstrom length resolution. *ACS Nano* **6**, 9237–9246 (2012).

79. A. Xomalis, R. Chikkaraddy, E. Oksenberg, I. Shlesinger, J. Huang, E. C. Garnett, A. F. Koenderink, J. J. Baumberg, Controlling optically driven atomic migration using crystal-facet control in plasmonic nanocavities . *ACS Nano* **14**, 10562–10568 (2012).

80. W. Zhu, R. Esteban, A. G. Borisov, J. J. Baumberg, Quantum mechanical effects in plasmonic structures with subnanometre gaps. *Nat. Commun*. **7**, 11495 (2016).

81. X. Zhang, B. Liu, C. Hu, S. Chen, X. Liu, J. Liu, F. Chen, J. Chen, F. Xie, A facile method in removal of PVP ligands from silver nanowires for high performance and reusable SERS substratet. *Spectrochim. Acta A Mol. Biomol. Spectrosc.* **228**, 117733 (2020).

82. J. He, S. Unser, I. Bruzas, R. Cary, Z. Shi, G. D. Boreman, R. Mehra, K. Aron, L. Sagle, The facile removal of CTAB from the surface of gold nanorods. *Colloids Surf. B Biointerfaces* **163**, 140–145 (2018).

83. M. Fan, A. G. Brolo, Factors that affect quantification in surface enhanced raman scattering. *ACS Nano* **19**, 3969–3996 (2012).

84. T. Matsuura, B. Slovick, Y. Shimoyama, Growth kinetics of self assembled monolayers of thiophene and terthiophene on Au(111): an infrared spectroscopic study. *Eur. Phys. J. E* **7**, 223–240 (2002).

85. A. Bard, R. Rondon, D. T. Marquez, A. E. Lanterna, J. C. Scaiano, How fast can thiols bind to the gold nanoparticle surface? *Photochem. Photobiol.* **94**, 1109–1115 (2018).
